# High concordance rate of capillary electrophoresis workflow for microsatellite instability analysis and mismatch repair (MMR) immunostaining in colorectal carcinoma

**DOI:** 10.1371/journal.pone.0284227

**Published:** 2023-04-25

**Authors:** Wenya Huang, Chung-Liang Ho, Chung-Ta Lee, Wan-Li Chen, Shu-Ching Yang, Nan-Haw Chow, Yi-Lin Chen

**Affiliations:** 1 Department of Pathology, Molecular Diagnosis Laboratory, National Cheng Kung University Hospital, Tainan, Taiwan; 2 Molecular Medicine Core Laboratory, Research Center of Clinical Medicine, National Cheng Kung University Hospital, Tainan, Taiwan; 3 Department of Medical Laboratory Science and Biotechnology, College of Medicine, National Cheng Kung University, Tainan, Taiwan; 4 Institute of Molecular Medicine, College of Medicine, National Cheng Kung University, Tainan, Taiwan; CNR, ITALY

## Abstract

Microsatellite instability (MSI) is the primary predictive biomarker for therapeutic efficacies of cancer immunotherapies. Establishment of the MSI detection methods with high sensitivity and accessibility is important. Because MSI is mainly caused by defects in DNA mismatch repair (MMR), immunohistochemical (IHC) staining for the MMR proteins has been widely employed to predict the responses to immunotherapies. Thus, due to the high sensitivity of PCR, the MSI-PCR analysis has also been recommended as the primary approach as MMR IHC. This study aimed to develop a sensitive and convenient platform for daily MSI-PCR services. The routine workflow used a non-labeling QIAxcel capillary electrophoresis system which did not need the fluorescence labeling of the DNA products or usage of a multi-color fluorescence reader. Furthermore, the 15 and 1000 bp size alignment markers were used to precisely detect the size of the DNA product. A cohort of 336 CRC cases was examined by MSI-PCR on the five mononucleotide MSI markers recommended by ESMO. The PCR products were analyzed in the screening gels, followed by high-resolution gel electrophoresis for confirmation if needed. In the MSI-PCR tests, 90.1% (303/336) cases showed clear major shift patterns in the screening gels, and only 33 cases had to be re-examined using the high-resolution gels. The cohort was also analyzed by MMR IHC is, which revealed 98.5% (331/336) concordance with MSI-PCR. In the five discordant cases, 4 (3 MSI-L and 1 MSS) showed MSH6 loss. Besides, one case exhibited MSI-H but no loss in the MMR IHC. Further NGS analysis, in this case, found that missense and frameshift mutations in the PMS2 and MSH6 genes occurred, respectively. In conclusion, the non-labeling MSI-PCR capillary electrophoresis revealed high concordance with the MMR IHC analysis and is cost- and time-effective. Therefore, it shall be highly applicable in clinical laboratories.

## Introduction

Colorectal carcinoma (CRC) is characterized as malignant growth inside the colon, rectum, or vermiform appendix. It is among the deadliest and most commonly diagnosed cancer in the world. Due to changes in lifestyles and diet habits in recent years, the incidence of CRC has been climbing [1]. In 2018, nearly 2 million new CRC cases were diagnosed, and about 1 million related deaths occurred [2]. The molecular features of CRC are associated with its clinicopathological patterns and treatment regimens [3]. CRC develops via two major pathways. Most CRCs follow the chromosomal instability pathway, characterized by gross chromosomal abnormalities, early mutations in the adenomatous polyposis coli gene, and microsatellite stable (MSS) status [4, 5]. The second type, involving approximately 15% of CRC cases, is characterized by defective mismatch repair (dMMR), which leads to the phenotype of genomic hypermutability, represented by microsatellite instability (MSI) [6].

Defects in MMR caused a 10- to 100-fold increase in the global mutation rates in colorectal mucosal cells [7]. The MMR system is a multiprotein system that acts as a proofreading machine to safeguard the replication fidelity through screening and directly repairing the mismatched nucleotides [7, 8]. The MMR system operates only when an error escapes the intrinsic error-checking mechanism of the DNA polymerase [7]. In human cells, the MMR system is composed of multiple interactive proteins, including MutS homologue (MSH) 2, 3, and 6, MutL homologue (MLH) 1 and 3, and PMS1 homologue (PMS) 1 and 2. These proteins work concertedly in sequential steps to perform repair of DNA mismatches [9].

Microsatellite instability (MSI) is involved in approximately 15% of sporadic CRCs and > 95% of hereditary nonpolyposis colorectal cancer (HNPCC), also known as Lynch syndrome [10]. Patients with Lynch syndrome mostly have a germline mutation in one of the MMR genes, such as *MLH1*, *MSH2*, *MSH6*, or *PMS2*, or an altered epithelial cell adhesion molecule (*EPCAM*) gene [11]. The 2019 National Comprehensive Cancer Network guidelines recommended that universal MMR or MSI testing be performed in all patients with a history of CRC for screening Lynch syndrome [12]. The MSI status is correlated with responses to anti-cancer therapies. Patients with the tumour stage 1 or 2 CRC involving the MSI pathway presented with lower response rates to the 5-fluorouracil chemotherapy [13]. However, the ones with high MSI showed better responses to the humanized programmed cell death-1 (PD-1) blocking antibodies than the ones otherwise [14]. In 2019, the European Society for Medical Oncology (ESMO) recommendations proposed the use of the dMMR immunohistochemistry (IHC) and MSI-PCR tests in recognition of sporadic cancers, including the colorectal, endometrial, small intestine, urothelial, central nervous system and sebaceous gland cancers, for the Lynch syndrome-like hypermutability characters [15–17]. And the use of multiple poly-A mononucleotide microsatellite (MS) markers was highly recommended for the MSI-PCR tests in reaching high sensitivities and specificities.

Though the MSI test has been the primary method to predict responses to PD1/PD-L1 therapies, recent clinical studies have found some CRC cases with MSI-H displayed unexpectedly ineffective to PD1/PD-L1 therapies due to inaccuracy or misinterpretation of the MSI-PCR test results before the treatments. These observations raised the potential necessity of performing both MSI-PCR and dMMR IHC analyses for the patients in evaluation for their benefits from the PD1/PD-L1 treatments. Thus, the concordance of the MSI-PCR and dMMR IHC test results remains to be evaluated [18, 19]. In this study, we developed a cost- and time-effective MSI test by non-labeling capillary electrophoresis [20]. Attributed to previous observations that these MS markers shifted mainly by more than ten base pairs in size, which shall be visualized by regular screening gel capillary electrophoresis (resolution 10–15 bp), we employed the QIAxcel DNA screening gel system as the primary approach to detect the shifting of MS markers [21–23]. The procedures are completed in approximately 20 minutes, significantly shorter than the fluorescence-based GeneScan analysis (running time ~ 2 hours), and do not require a fluorescence detection facility, therefore significantly lowering the cost of setup of the analysis device [24]. The MSI-PCR tests established here revealed high concordance with dMMR IHC analysis. The MSI-PCR platform established for the analysis of CRC here is also readily applicable for analyzing the MSI phenotypes in other types of cancer where the PD-1/PD-L1 immunotherapies are feasible [20, 25, 26].

## Methods

### Cohort

The cohort included 336 patients who had received surgery or adjuvant chemotherapies for CRC at the National Cheng Kung University Hospital (NCKUH) between January 2015 and December 2017 (Table 1). This study was approved by the Institutional Review of Board of National Cheng Kung University Hospital, Tainan, Taiwan. In study design, CRC cases diagnosed in National Cheng Kung University Hospital (NCKUH) between 2012 and 2020 were retrospectively collected with concurrent analysis of MMR status by PCR and IHC methods according to approved protocol (B-ER-109-152). There were 43 dMMR CRCs and 293 pMMR CRCs. One case was subjected to MMR gene analysis using next generation sequencing (NGS) (B-ER-108-311). The written informed consent was obtained from the participating subject with discrepancy between pMMR IHC and MSI-PCR. Principle investigators provide documentations of informed consent for medical records to be used in the present study. All patients received standard treatments and follow-up at NCKUH for colorectal cancers. All CRC cases were diagnosed by medical imaging evaluation and biopsy histopathologic reviews by two pathologists independently. The tumour/node/metastasis staging and histologic grading followed the American Joint Committee on Cancer (AJCC) classification.

**Table 1:** Clinicopathologic features of colorectal cancer cases in this study (N = 336).

	MSI-H		MSS+MSI-L		*p*-value
	n = 40	%	n = 296	%	
Mean age at diagnosis ±SD, years	59.8±13.3		60.4±13.2		
Gender					
Male	19	47.5	171	57.8	0.237
Female	21	52.5	125	42.2	
Location					
Proximal colon	27	67.5	86	29.1	<0.001
Distal colon	13	32.5	210	70.9	
Histologic type					
Adenocarcinoma	25	62.5	276	93.2	<0.001
Mucinous adenocarcinoma	15	37.5	15	5.1	
NA			5	1.7	
Histological feature					
Well	6	15.0	19	6.4	0.001
Moderate	26	65.0	247	83.4	
Poor	8	20.0	17	5.7	
NA			13	4.4	
TNM stage					
0-I	7	17.5	10	3.4	<0.001
II	17	42.5	31	10.5	
III	13	32.5	136	45.9	
IV	3	7.5	116	39.2	
NA			3	1.0	
T stage					
1	3	7.5	5	1.7	0.223
2	4	10.0	24	8.1	
3	21	52.5	150	50.7	
4	11	27.5	55	18.6	
NA	1	2.5	62	20.9	
N stage					
0	24	60.0	50	16.9	<0.001
1	6	15.0	108	36.5	
2	9	22.5	76	25.7	
NA	1	2.5	62	20.9	
M stage					
0	36	90.0	177	59.8	0.021
1	3	7.5	57	19.3	
NA	1	2.5	62	20.9	
*KRAS*					
Wild-type	11	27.5	161	54.4	0.801
Mutant	6	15.0	116	39.2	
NA	23		19	6.4	
*NRAS*					
Wild-type	17	42.5	262	88.5	1.000
Mutant	0	0.0	8	2.7	
NA	23	57.5	26	8.8	
*BRAF*					
Wild-type	28	70.0	134	45.3	<0.001
Mutant	12	30.0	10	3.4	
NA			152	51.4	

NA: not available.

### Immunohistochemistry (IHC)

IHC was performed using an immunostainer (automated slide staining instrument, *Ventana* Medical Systems). The monoclonal antibodies, pre-conjugated with biotin, were PMS-2 (AB: Ventana, Clone EPR3947), MLH-1 (AB: Ventana, Clone M1), MSH-6 (AB: Cell Marque, Clone: 44), and MSH-2 (AB: Cell Marque, Clone: 44). The IHC signal was visualized using the streptavidin–biotin complex method. All of the MMR IHC stains were independently reviewed by two pathologists (Lee C. T. and Chow N.H.), based on the revised Bethesda guidelines described previously [27]. Essentially, the signal was determined “loss” (i.e. abnormal) when their nuclear staining in tumor cells were absent in the presence of positive staining in the surrounding stromal cells. Weak but unequivocally positive signal was determined as “positive” (i.e. normal) expression.

### MSI-PCR test

**Fig 1:**
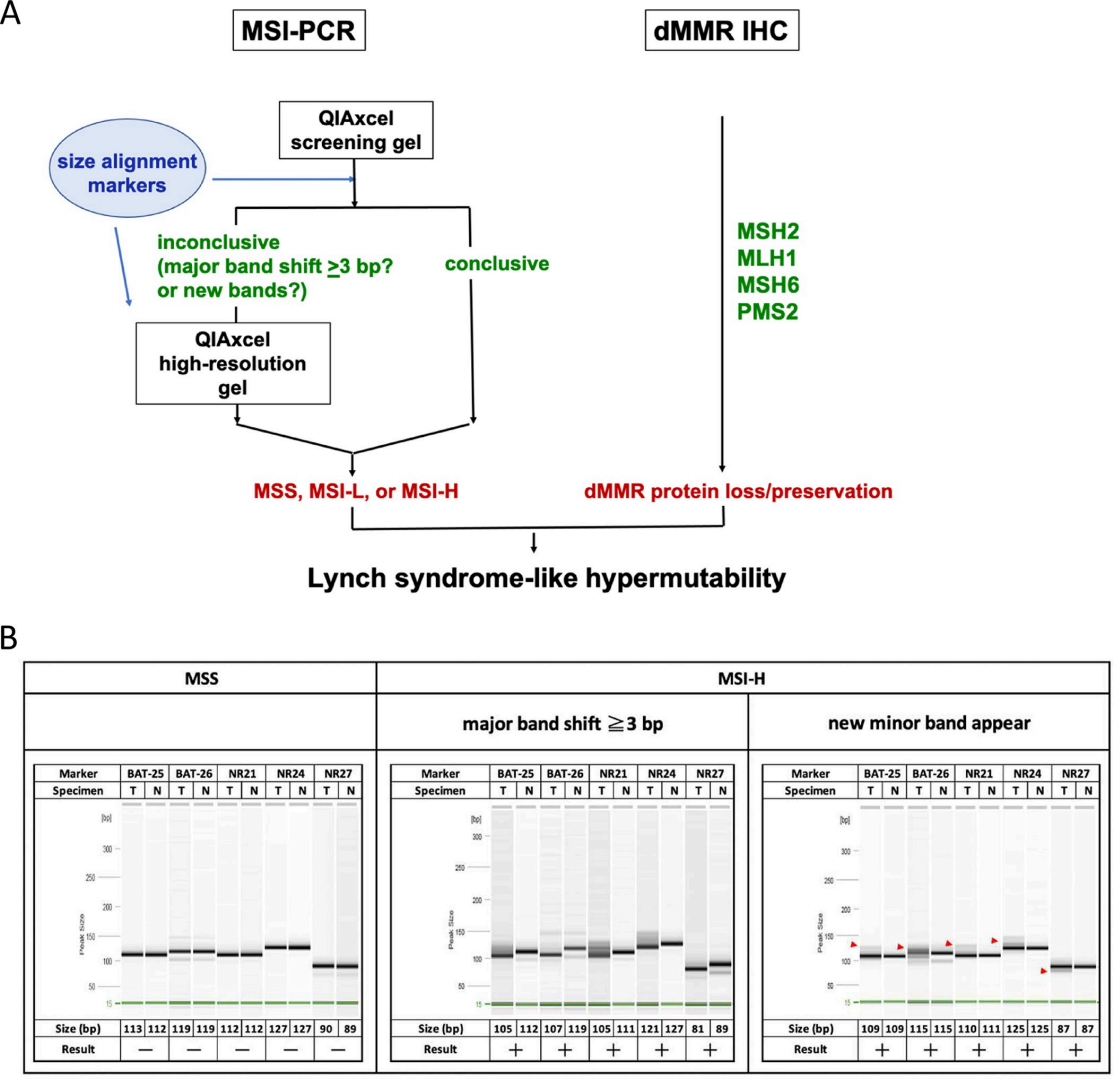
The MSI-PCR test developed in this study. **A Workflow of microsatellite PCR analysis.** The MSI-PCR test contains five poly-A mononucleotide MS markers. A marker was defined unstable in the tumour if the shift was equal to or larger than 3 bp in the major band or if new minor bands were present compared with those in the corresponding non-tumourous tissue. For the ones showing an inconclusive smearing pattern, the PCR products were re-run in a high-resolution gel to visualize the PCR bands more clearly. MSI-H: two or more of the five microsatellite markers show instability. MSI-L: one of the five microsatellite markers shows instability. MSS: no markers show instability. **B Representative images of the MSI-PCR gel electrophoresis.** The left panel, the MSS result. The middle panel, an MSI-H result with the major DNA products shifted by 3 bp or larger in the tumour. The sizes of the major DNA products are shown on the bottom of the gel images. The right panel, an MSI-H result with presentation of new minor DNA bands in the tumour, as compared with the non-tumourous region. T, tumour. N, non-tumourous region.

DNA from tumour or tumour-free tissue was extracted using the *Qiagen* QIAamp *DNA FFPE* Tissue DNA extraction kit (Qiagen, Hilden, Germany). In the absence of peri-tumourous tissue in the case, a blood sample was used instead for the tests. In total, 30 peri-tumorous specimens were replaced by blood of the same individuals. Protocol of the PCR reactions followed that described previously [28]. The PCR products were analysed through capillary electrophoresis using the QIAxcel DNA screening kit and *Advanced System* (*Qiagen* GmbH, Hilden, Germany) according to the manufacturer’s instructions. The PCR products which showed inconclusive smearing patterns in the screening gel were re-run in a high-resolution gel by using the *QIAxcel Advanced System* according to the manufacturer’s instructions (Fig 1A) [29].

Based on the 1997 Bethesda Guideline, MSI-H was defined as instability at two or more of the five MS markers and MSI-L as instability at one locus. In all other instances, tumours were considered MSS. The 15 and 1,000 bp size alignment markers were used to precisely detect the size of the DNA product. Each DNA alignment marker had to appear as a single peak with no shoulder appearing in the *QIAxcel Advanced System*. Alignment of the size markers among various lanes preceded the size determination of the PCR product (S1 Fig). A marker was considered positive (unstable) if the shift was equal to or larger than 3 bp in the major band or if new minor bands were present compared with those for the non-tumourous tissue (Fig 1B) [30].

### Determination of the sensitivities of the MSI-PCR analysis

Sensitivities of the MSI analysis using capillary electrophoresis were determined using genomic DNA of the HCT116 cells or the MSI-H FFPE tissues, pre-mixed with various amounts of normal fresh frozen or formalin-fixed paraffin-embedded (FFPE) DNA to 30 ng total in each reaction. The HCT116 cell was heterozygous in *MLH1* c.755C>A and deficient in MMR function [31]. Samples containing 100%, 50%, 20%, 10%, 7.5%, 5%, and 2.5% HCT116 DNA were detected in the MSI-PCR tests.

### Next generation sequencing of the MMR genes

For the CRC case which showed MSI-H in the MSI-PCR test but normal MMR protein signals in the dMMR-IHC analysis, further NGS analysis was performed. The Human Colorectal Cancer QIASeq Targeted DNA Panel was employed (Qiagen). The gene coding regions and the essential splice sites were detected. Amplicons were dual barcoded for sample identification. The DNA libraries were sequenced using 150-bp paired-end reads in an Illumina MiSeq sequencer. Mean sequence read depths of 387× and 443× were obtained for the tumour tissue and blood, respectively. NGS data were analysed using the GeneGlobe Data Analysis software for variant calling. Analysis was focused on the Lynch syndrome-associated gene variations in the MMR genes *MSH2*, *MSH6*, *PMS2*, and *MLH1*, as well as those in the *ATM*, *BRCA1*, *BRCA2*, *CHEK2*, *PALB2*, and *TP53* genes, based on the National Comprehensive Cancer Network (NCCN) Clinical Practice Guidelines in Oncology (2020). Variants were classified as pathogenic or likely pathogenic based on the allele frequency (>5%), the effect on protein function, and ClinVar Assertions (NIH, USA).

### Statistical analysis

In our CRC cohort, the various clinicopathologic factors and molecular markers were compared through the Fisher’s exact test by using the SPSS statistical software package. SPSS 17.0 for Windows (SPSS Inc., Chicago, IL) was used for all analyses. Significance was set at *p* < 0.001 (two-sided).

## Results

**Fig 2:**
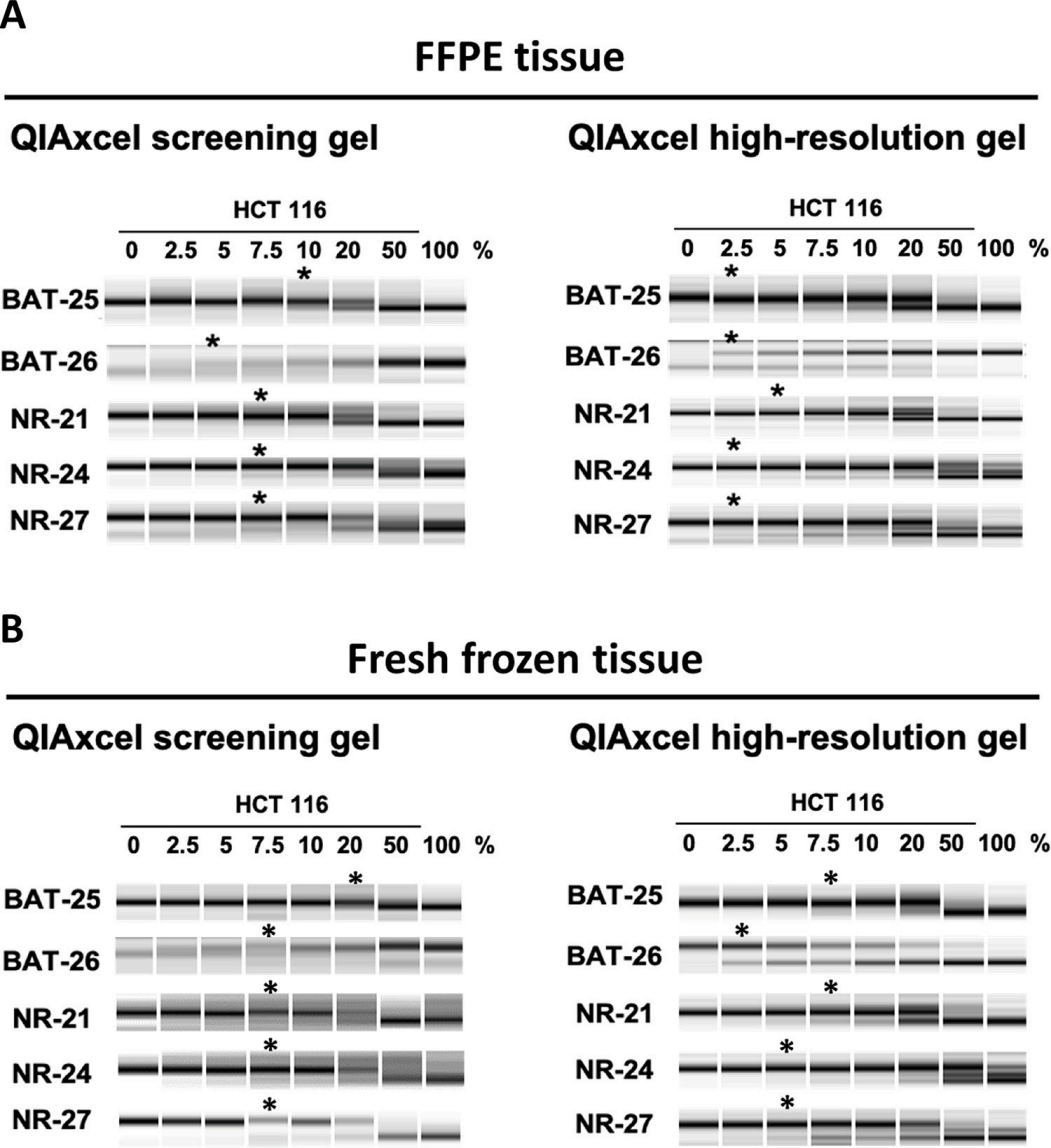
Sensitivities of the MSI-PCR tests using the screening and high-resolution gel capillary electrophoresis. Genomic DNA of the MSI (+) HCT116 cell line was mixed with that of normal FFPE tissue (A) and fresh frozen tissue (B) in various ratios for the MSI-PCR analysis. The PCR products were visualized by the QIAxcel screening (left) and high-resolution (right) gel electrophoresis for determination of the respective limit of detection (LOD). * indicates the LOD value for each MS marker.

We established the MSI-PCR workflow using an MS panel containing five poly-A mononucleotide markers. A marker was defined as unstable in the tumour if the shift was equal to or larger than three bp in the major band or if new minor bands were present compared with those in the corresponding non-tumourous tissue. For the ones showing inconclusive smearing patterns in the screening gel, the PCR products were re-run in a high-resolution gel with a resolution of 1 to 3 bp to visualize the PCR bands more clearly (Fig 1A). Based on this criterion, this test’s detection of limit (LOD) was evaluated. The genomic DNA of the MSI (+) HCT116 cell line was mixed with that of normal FFPE or fresh frozen tissues in various ratios for the analysis. The results showed that in the case of FFPE DNA specimens, the LOD for different MS markers was 5% for BAT-26, 10% for BAT-25, and 7.5% for NR-21, NR-24, and NR-27 by using the screening gel. When using the high-resolution gel, the LOD was 2.5% for BAT-26, BAT-25, NR-24, and NR-27, and 5% for NR-21 (Fig 2A). In the fresh frozen tissue DNA, the LODs for various MS markers were close to that in the FFPE DNA (Fig 2B). These findings indicate that the use of the high-resolution gel revealed considerably higher sensitivity, and it is recommended for the specimens presenting with MSI alleles lower than 10% or so. The performance of the method established in this study was also equivalent to that of the fluorescence-based GeneScan approach previously reported, suggesting that it is an economical method readily applicable to laboratories with no access to a fluorescence detection facility (S2 Fig) [32]. As expected, 12 CRC specimens were examined by both the GeneScan and MSI-PCR analyses and showed 100% concordance between the results revealed by the two approaches (S1 Table and S2 Fig). Furthermore, the MSI-PCR tests using the non-labeling capillary system (QIAxcel) developed in this study have consistently passed the proficiency tests in the College of American Pathologists (CAP) since 2018 and been accredited by the Taiwan Accreditation Foundation (TAF; ISO15189) for approval in use in clinical laboratories.

Using the MSI-PCR method established in this study, we analyzed the MSI status in a cohort of 336 CRC cases in Taiwan. Among them, 303 (90%) cases presented with clear major shift patterns in the screening gels, and only 33 had to be re-examined by the high-resolution gel analysis, which suggested that the screening gel system was able to identify the majority of the MSI changes in the CRC tumours. The results indicated that 12% (40/336) presented with MSI-H, similar to the findings previously reported in other world areas [33, 34]. Interestingly, the MSI-H tumours were prevalent in the proximal colon (27/40, 68%), unlike MSI-L/MSS tumours, which are prevalent in the distal colon (210/296, 71%), indicating that the MSI phenotype was likely associated with tumour location in CRC [35, 36].

**Fig 3:**
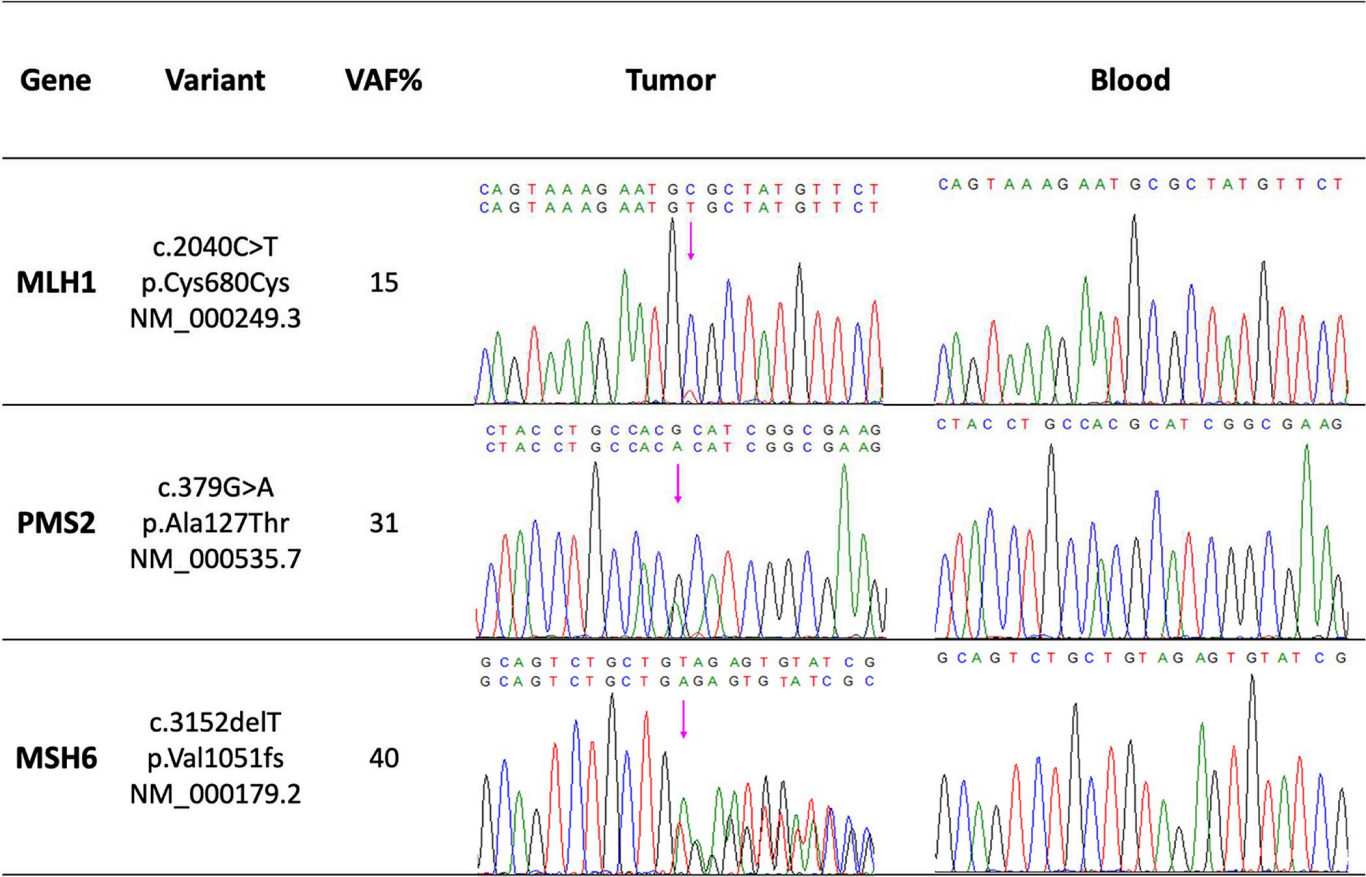
Analysis of the MMR gene variants by NGS and Sanger sequencing in the discordant case showing discordance in MSI-PCR and dMMR IHC tests. The NGS analysis of the MMR and oncogene panel in the tumour and corresponding blood specimens were analysed by NGS. The genetic variants identified by NGS were confirmed by Sanger sequencing. VAF%, variant allele frequency.

The concordance of the MSI-PCR and dMMR IHC, which detects the MSH2, MLH1, PMS2, and MSH6 proteins, was evaluated. Data summarized in Table 2 show that the concordance between the two approaches was 98.5% (331/336). Among the five discordant cases, four (three MSS and one MSI low, identified by both the screening and high-resolution gel systems) showed MSH6 loss in the IHC analysis. Because MSH6 is mainly involved in repairing DNA mismatch rather than insertion/deletion, its deficiency was expected to reveal no significant shifting of MS markers in the MSI PCR analysis [37]. Therefore, the MSI PCR method developed in this study sensitively detected the size shifts of MS markers in our subjects. In addition, one case exhibited MSI-H but no loss in the dMMR IHC. Results of the NGS analysis, in this case, showed that, as compared with the non-tumourous region, the CRC tumour specimen harbored three major somatic mutations: 15% alleles with *MLH1* c.2040C>T silent mutation (NM_000249.3), 31% with *PMS2* c.379G>A, which results in missense mutation Ala127Thr (NM_000535.7), and 40% with *MSH6* c.3152delT mutation, which results in a frameshift at Val1051 and premature translation termination (NM_000179.2) (Fig 3). All mutations in the tumour specimens were confirmed using Sanger sequencing and not detected in the patient’s blood sample. Based on these results, it was speculated that the *MSH6* c.3152delT p.Val1051fs induced premature translation termination might result in an aberrant protein product, which interferes with the normal function of the dMMR machinery and leads to MSI.

**Table 2:** Concordance between MSI-PCR and dMMR IHC analyses.

MSI PCR (n = 336)	MMR IHC		
	Preserved	Loss	Consistant
MSS+MSI-L	292	4	98.5%
MSI-H	1	39	

## Discussion

Tumour hypermutability has been recognized as a typical phenotype in many types of cancer and found to serve as an important biomarker for cancer subtyping, therapies, and prognosis in recent years [38]. The MSI status has been used as the critical predictive biomarker for response to PD-1/PD-L1 immunotherapies for many highly prevalent cancers. However, the characterization of the MSI status in most types of cancer has not been comprehensively investigated. A reliable and easy-to-perform MSI test is inevitable for evaluating the feasibility of immunotherapies for patients in need.

It has been a big concern that results of the MSI-PCR and dMMR IHC tests often showed a discrepancy in determining the Lynch syndrome-like hypermutability phenotype in the tumour [38, 39]. The current study developed a time- and cost-effective approach for the MSI-PCR test that does not require access to a fluorescence-based GeneScan facility. The current method employs a one-step non-labeling capillary electrophoresis system, which completes the DNA analysis in approximately 15 to 20 min, much faster than the GeneScan analysis, which usually takes a few hours. Using the size alignment markers to ensure that the distances by which DNA products migrate in the gel solely depend on their sizes, the measurement of the size of each DNA product can be precise. With the aid of these size alignment markers in size determination of the MS PCR products, the MSI-PCR platform set up in this study revealed a high concordance rate of 98.5% with the dMMR IHC in terms of determining the Lynch syndrome-like hypermutability phenotype, decreasing the situation of the MSI-PCR vs. IHC discordance reported in many other studies [39, 40].

Comprehensive studies of the association of MSI with cancer subtyping and prognosis remain to be investigated [25, 40]. We found that the MSI-H was more prevalent in proximal CRC than the MSI-L/MSS was (68% vs. 29%), suggesting MSI is unlikely related to the tissue but to the tumour that develops on it. It has been reported that CRC in the proximal and distal colon presents many different genetic and morphological characteristics, including embryonic origin, blood supply, innervation, lymphatic drainage, and lumen environment [35, 36]. The proximal, i.e., right-sided colon carcinoma (RCC), is also associated with poor differentiation and a worse prognosis [41]. It is not clear whether MSI is associated with these phenotypic characteristics. Nevertheless, some studies have shown that Lynch syndrome-associated CRC was likely to correlate with mild disease progression and better prognosis [33, 34]. Therefore, whether the MSI status is associated with various clinicopathological characteristics related to CRC tumour locations remains to be investigated. In the CRC cohort of this study, we also found that the MSI-H cases harbored higher *BRAF* mutation rates than the MSI-L/MSS cases (30% vs. 6.9%) but not *KRAS* or *NRAS* mutations. Previous studies have reported that the *BRAF* mutation was prevalent in sporadic MSI-H and the CpG island methylator phenotype (CIMP) CRC, where *MLH1* promoter hypermethylation is often seen, indicating that genomic instability in these CRCs promotes *BRAF* mutation, which plays a vital role in initiating tumourigenesis [42, 43]. The clinical significance of the combination of the MSI and *BRAF* mutation in CRC progression shall be elaborated. Thus, the MSI-H status contributes to the neoantigen production and stimulation of T-cell immune surveillance, leading to favorable responses to PD-1/PD-L1 immune therapies [16, 44]. Moreover, a recent study of the NGS-based MSI test in CRC has shown a strong concordance with the MSI-PCR test using a five-mononucleotide MS panel, indicating the high sensitivity of the MSI-PCR test [45]. With its cost and time effectiveness, the MSI PCR test can be widely applied in identifying cancers with dMMR phenotype.

In summary, this study developed a time- and cost-effective protocol for the MSI-PCR platform, which provides sensitive detection of the size shifts of the MS markers and determines the MSI status as a predictive response biomarker to cancer immunotherapies. With this detection method, the concordance of the MSI-PCR and dMMR IHC in evaluating the genomic hypermutability phenotype reaches 98.5%, which is likely satisfactory for CRC. Furthermore, the platform established in this study shall be readily applicable to MSI detection in other types of cancer which are in the intensive investigation for immune therapies.

## Supporting information

S1 Table MSI status defined by the five mononucleotides repeat markers of CRC cases analyzed using MMR-IHC, GeneScan and MSI-PCR. (XLS)

S1 Fig QIAxcel advanced system. Alignment of the size markers among various lanes preceded the size determination of the PCR product.
(TIF)

S2 Fig Analysis of the MSI PCR products by QIAxcel and GeneScan methods. (TIF)

S1 Raw images (PDF)
