# Supplementary figures and images for: High concordance rate of capillary electrophoresis workflow for microsatellite instability analysis and mismatch repair (MMR) immunostaining in colorectal carcinoma
Source: PLoS One. 2023 Apr 25;18(4):e0284227. doi: 10.1371/journal.pone.0284227 (PMC10128978; doi:10.1371/journal.pone.0284227)

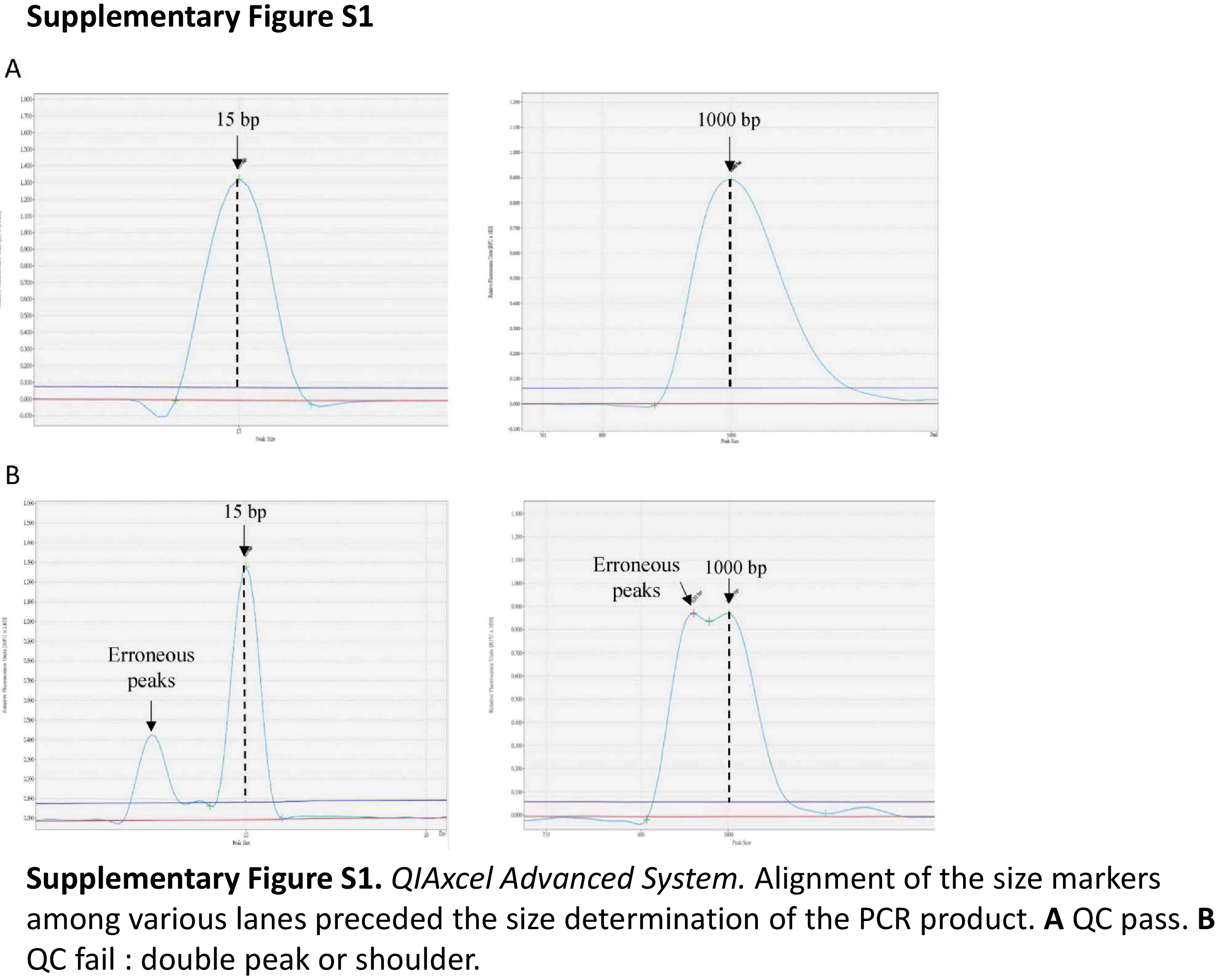

Supplement: S1 Fig — Alignment of the size markers among various lanes preceded the size determination of the PCR product. (TIF) [file pone.0284227.s002.tif]

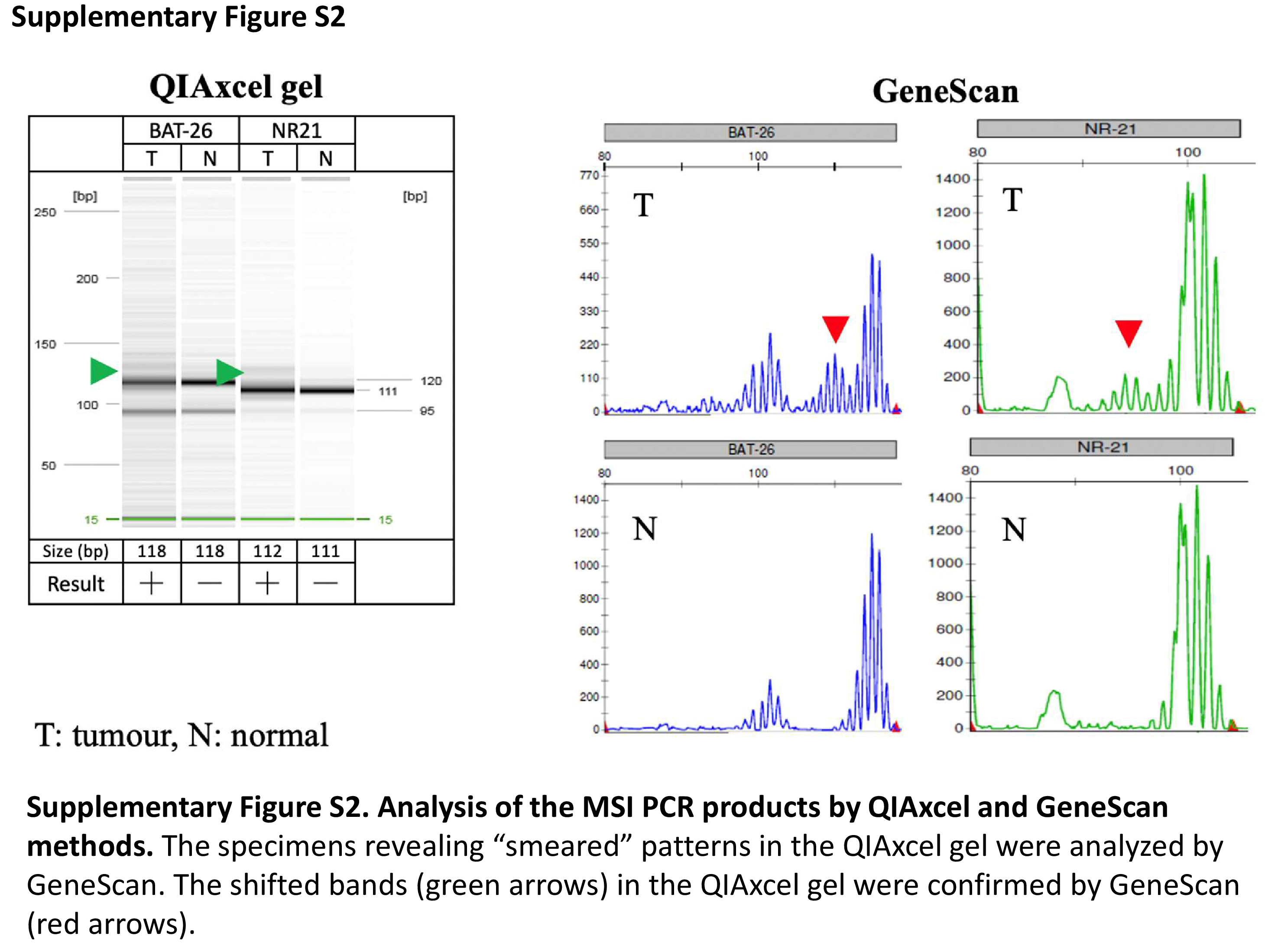

Supplement: S2 Fig — (TIF) [file pone.0284227.s003.tif]
