# Supplementary material for: High concordance rate of capillary electrophoresis workflow for microsatellite instability analysis and mismatch repair (MMR) immunostaining in colorectal carcinoma
Source: PLoS One. 2023 Apr 25;18(4):e0284227. doi: 10.1371/journal.pone.0284227 (PMC10128978; doi:10.1371/journal.pone.0284227)
Supplement: S1 Raw images — (PDF) [file pone.0284227.s004.pdf]

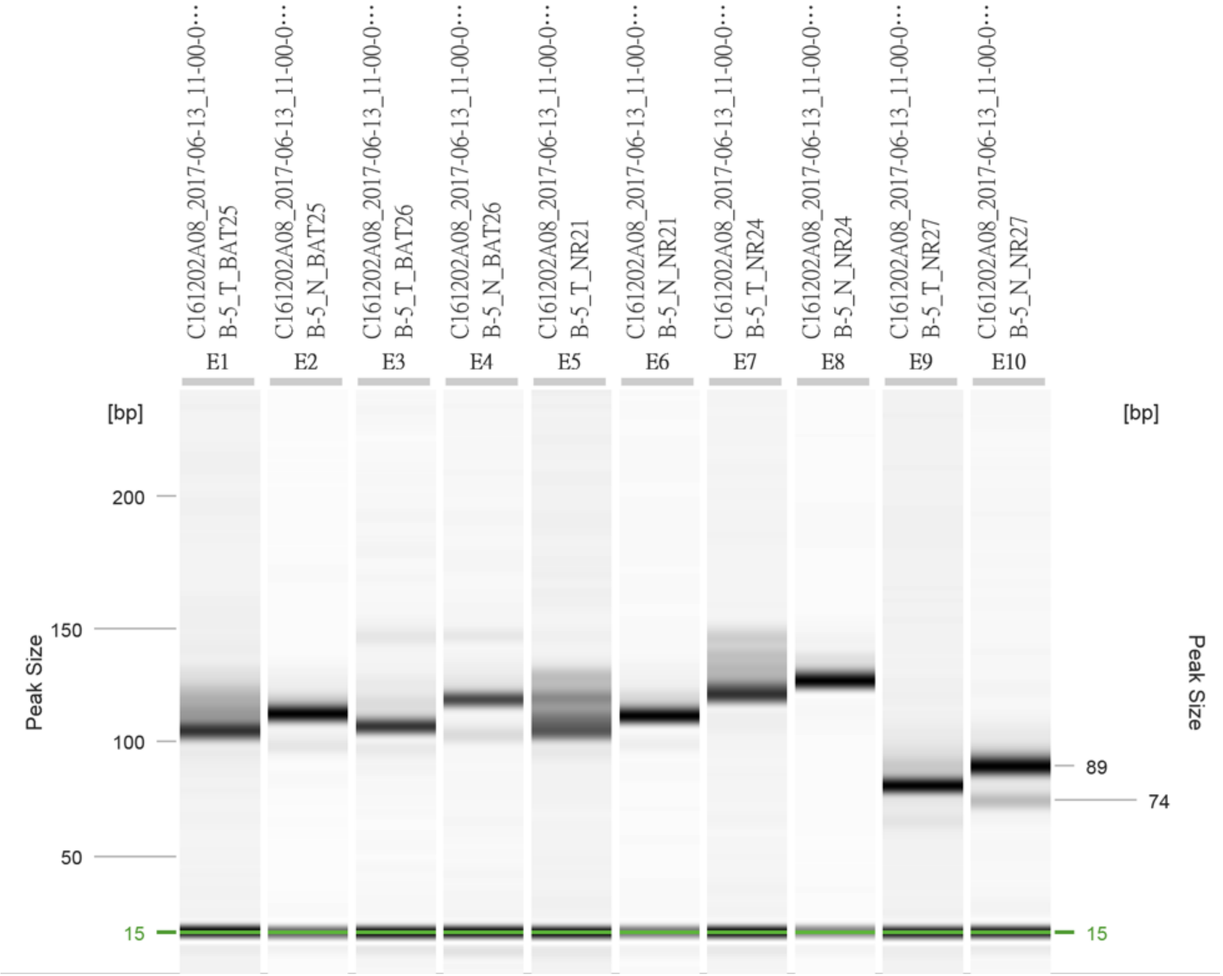

Figure 1B MSS original image

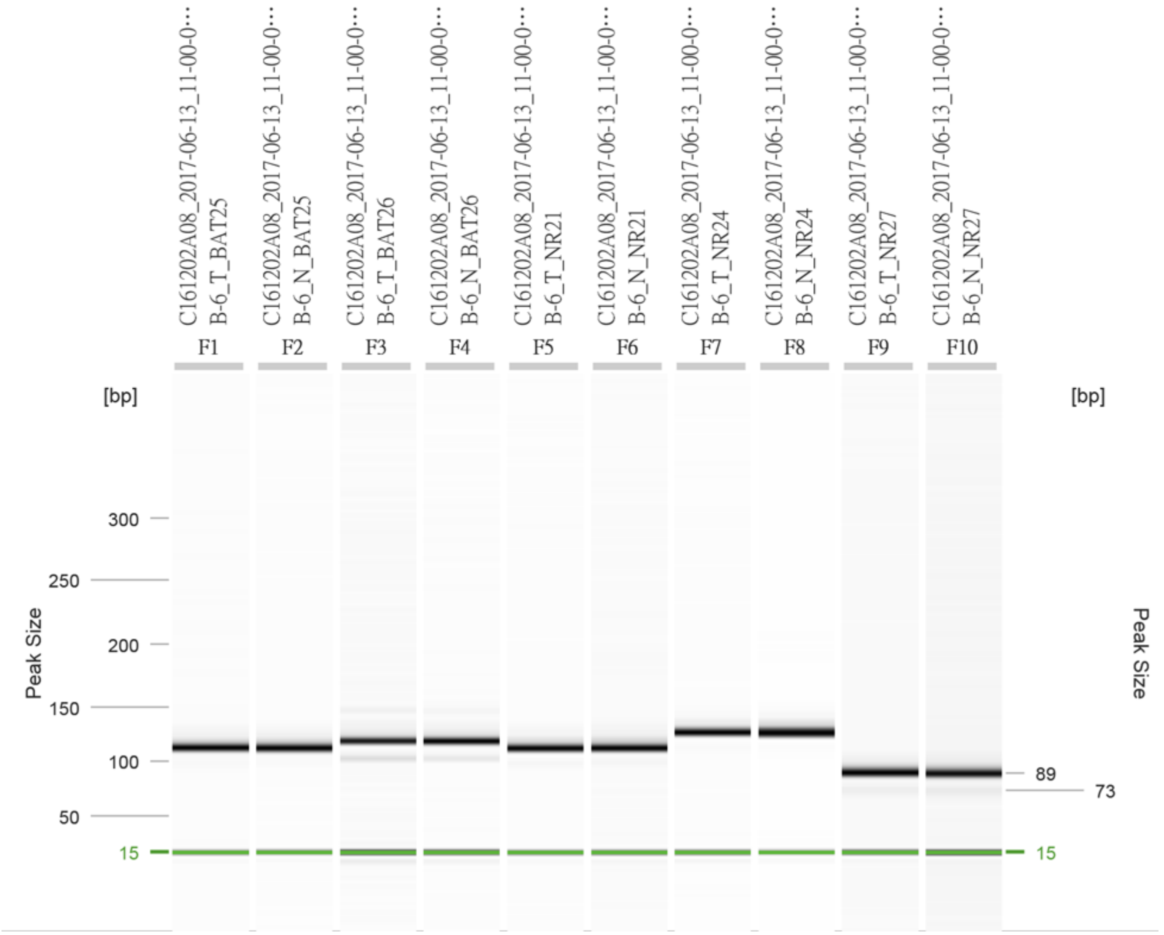

Figure 1B MSI-H original image (major band shift) original image

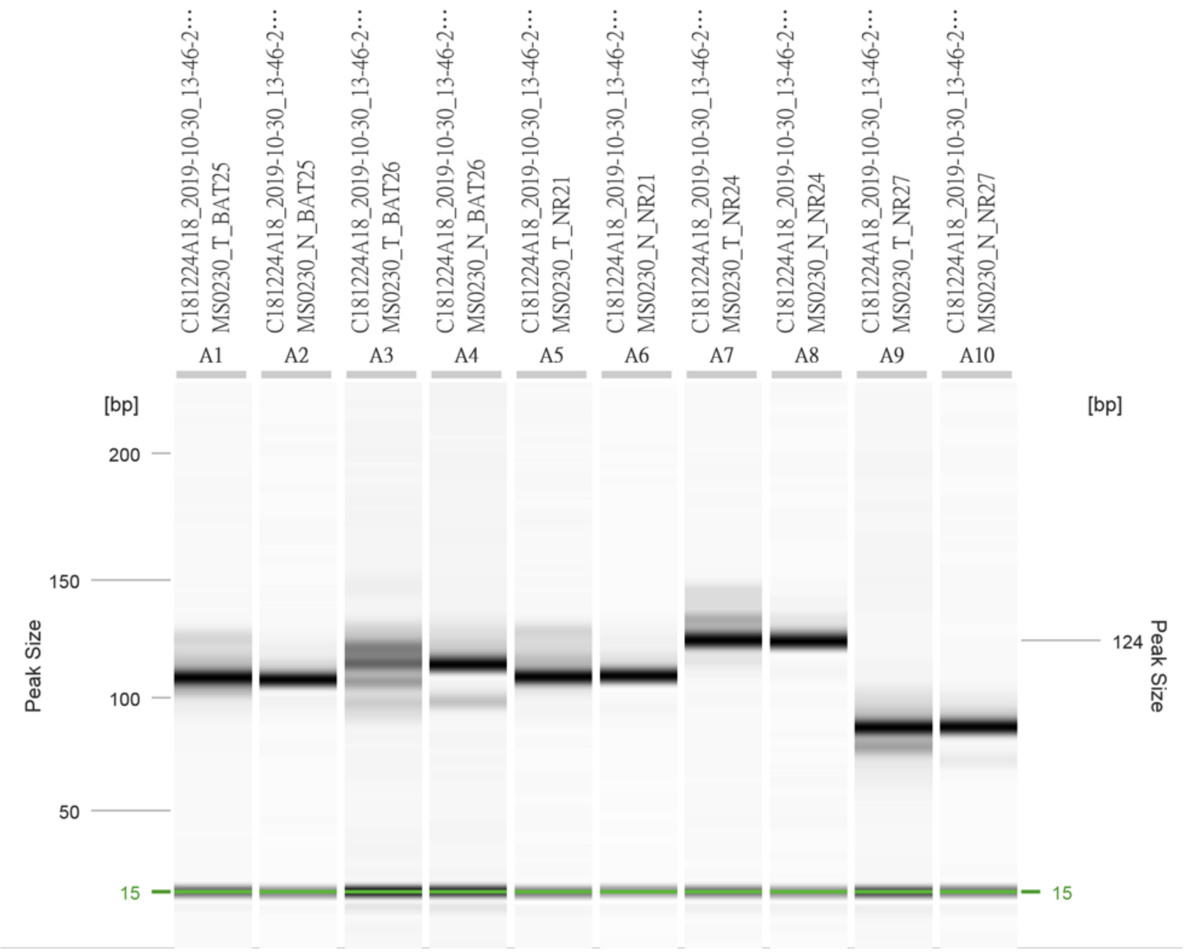

Figure 1B MSI-H original image (new minor band) original image

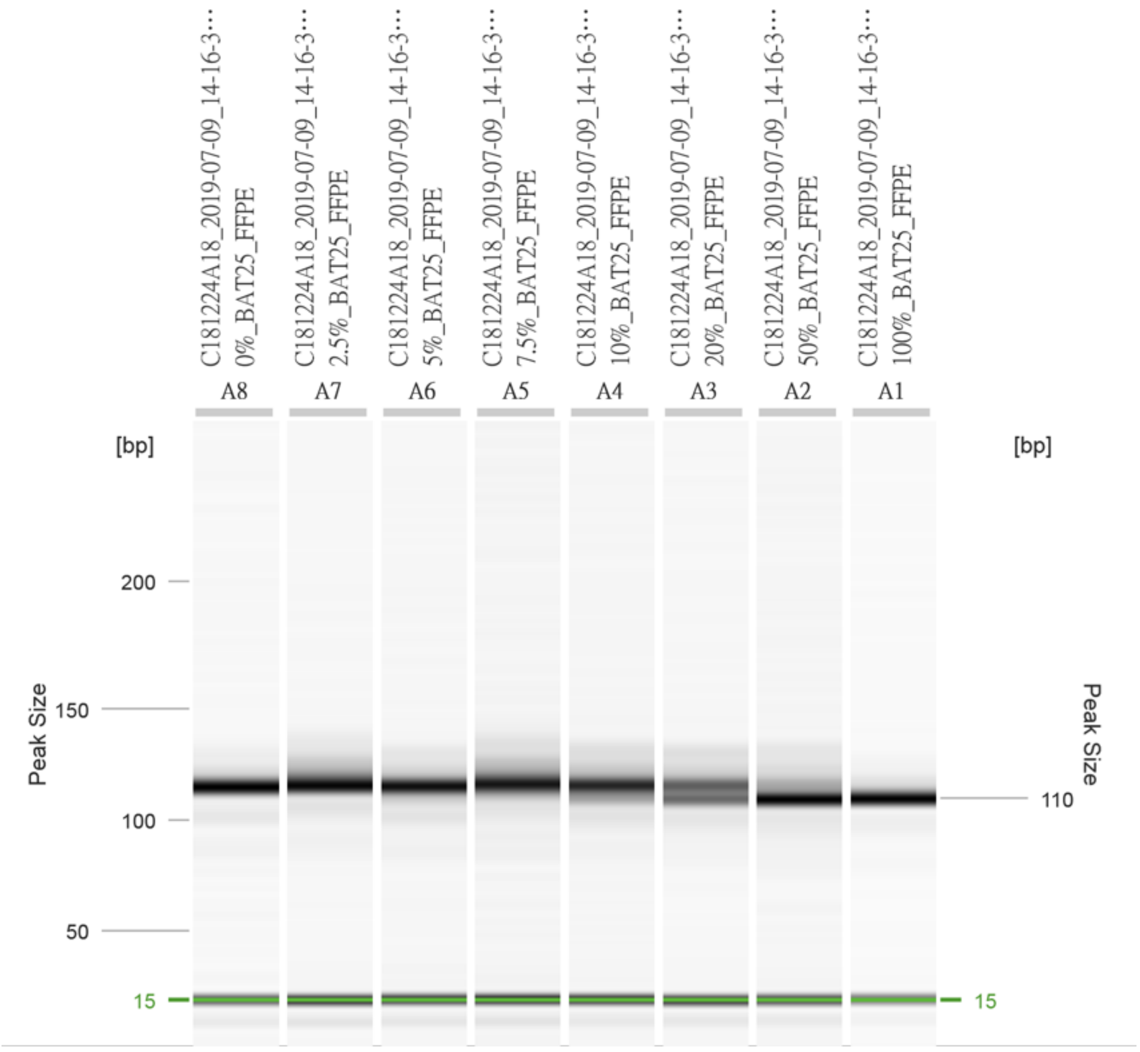

Figure 2 A FFPE BAT-25 Screen gel original image (Left side)

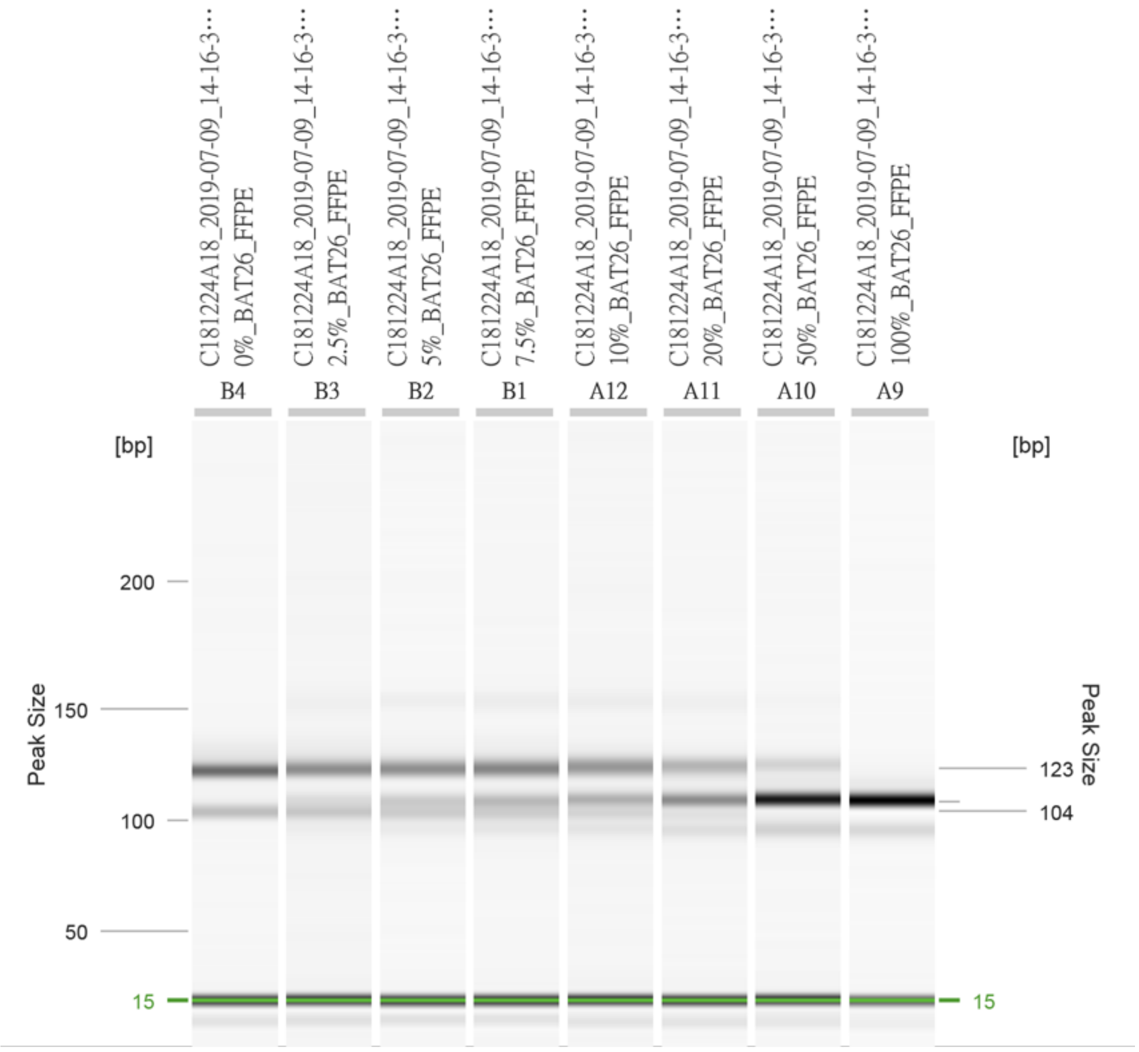

Figure 2 A FFPE BAT-26 Screen gel original image (Left side)

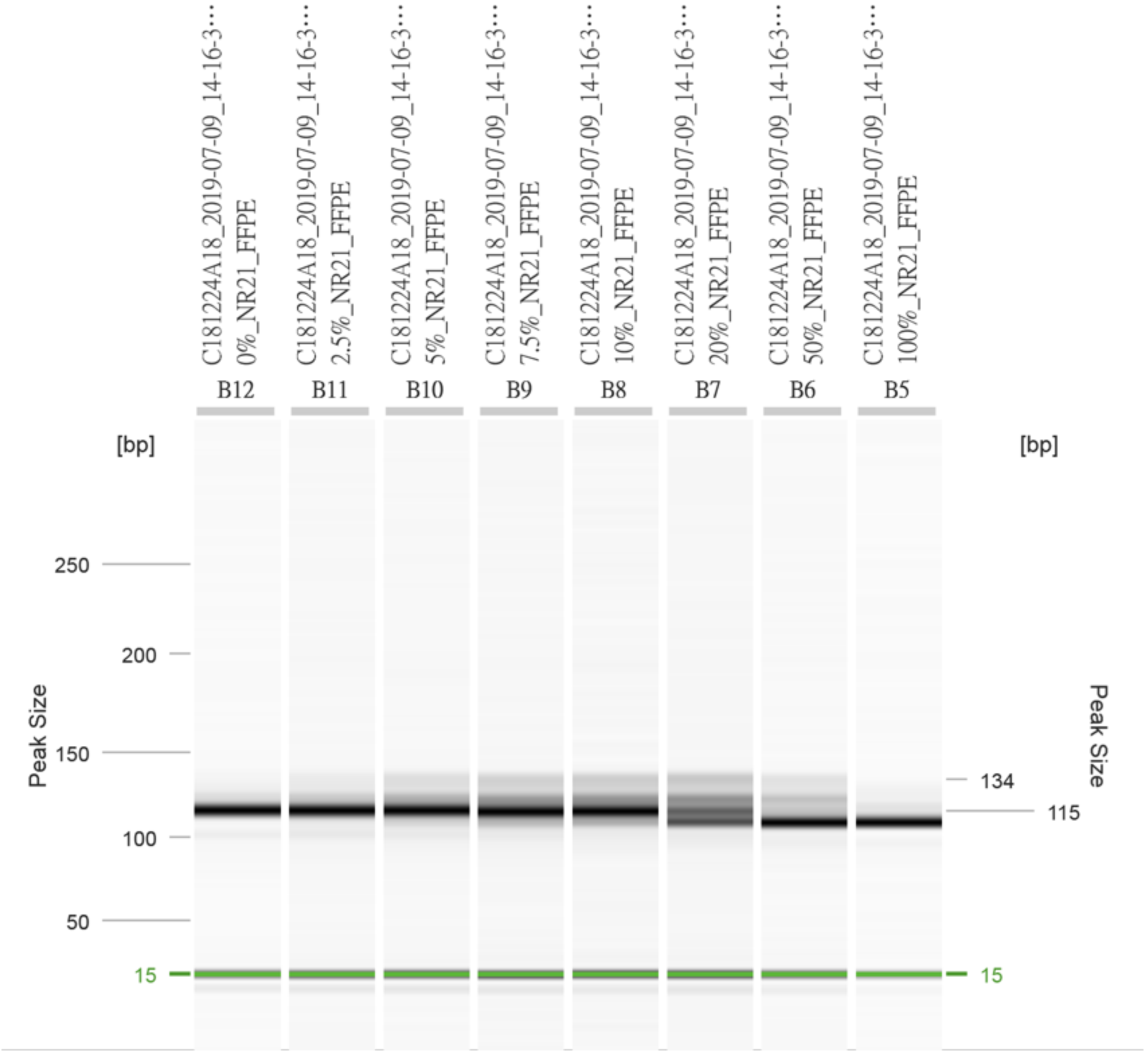

Figure 2 A FFPE NR-21 Screen gel original image (Left side)

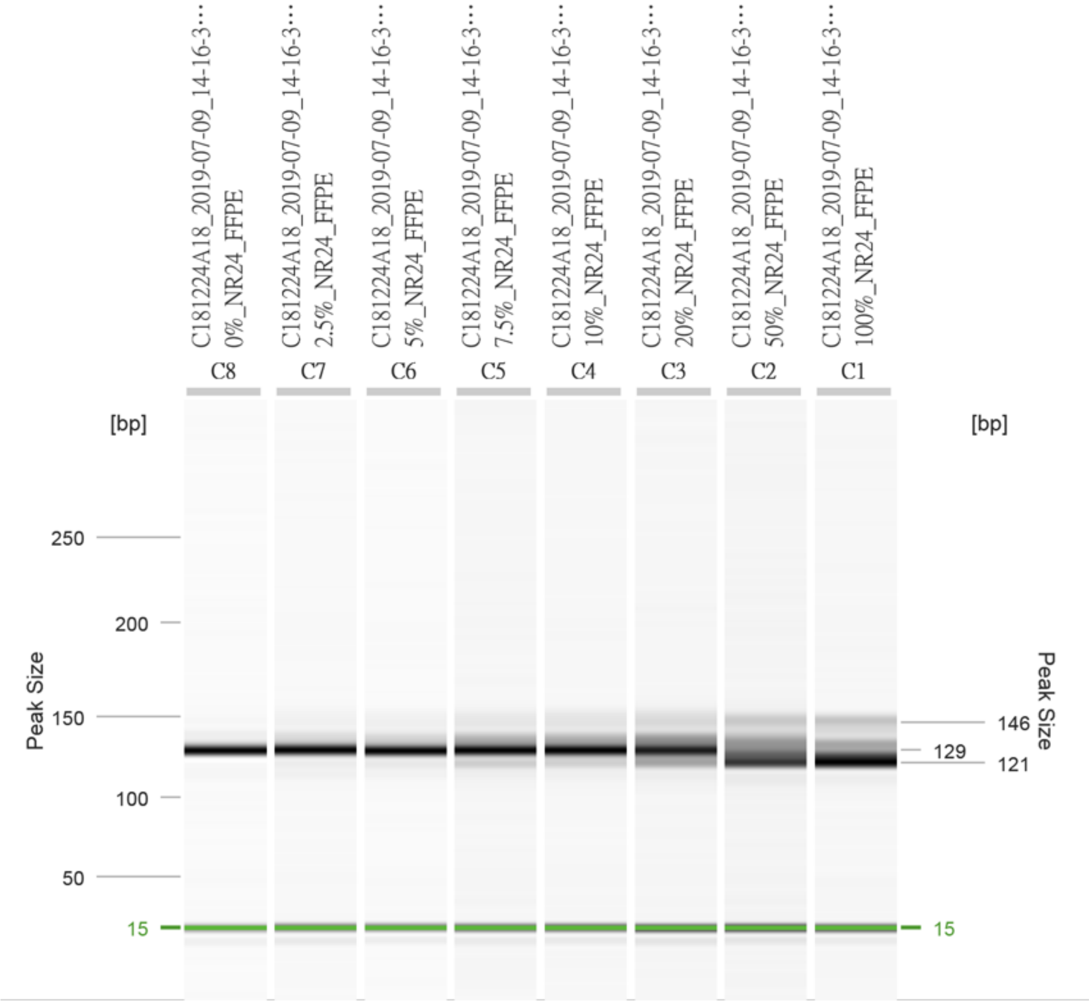

Figure 2 A FFPE NR-24 Screen gel original image (Left side)

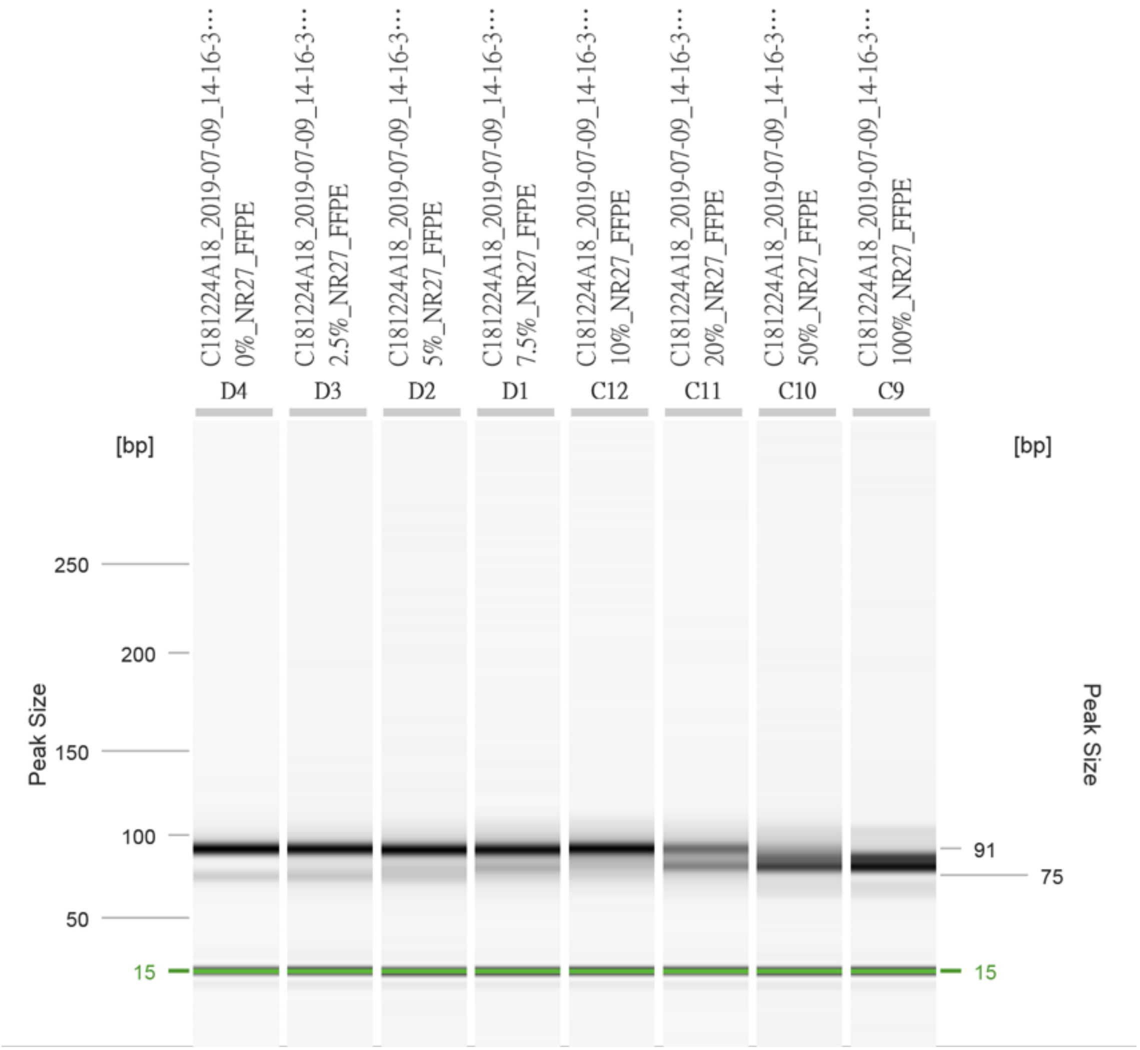

Figure 2 A FFPE NR-27 Screen gel original image (Left side)

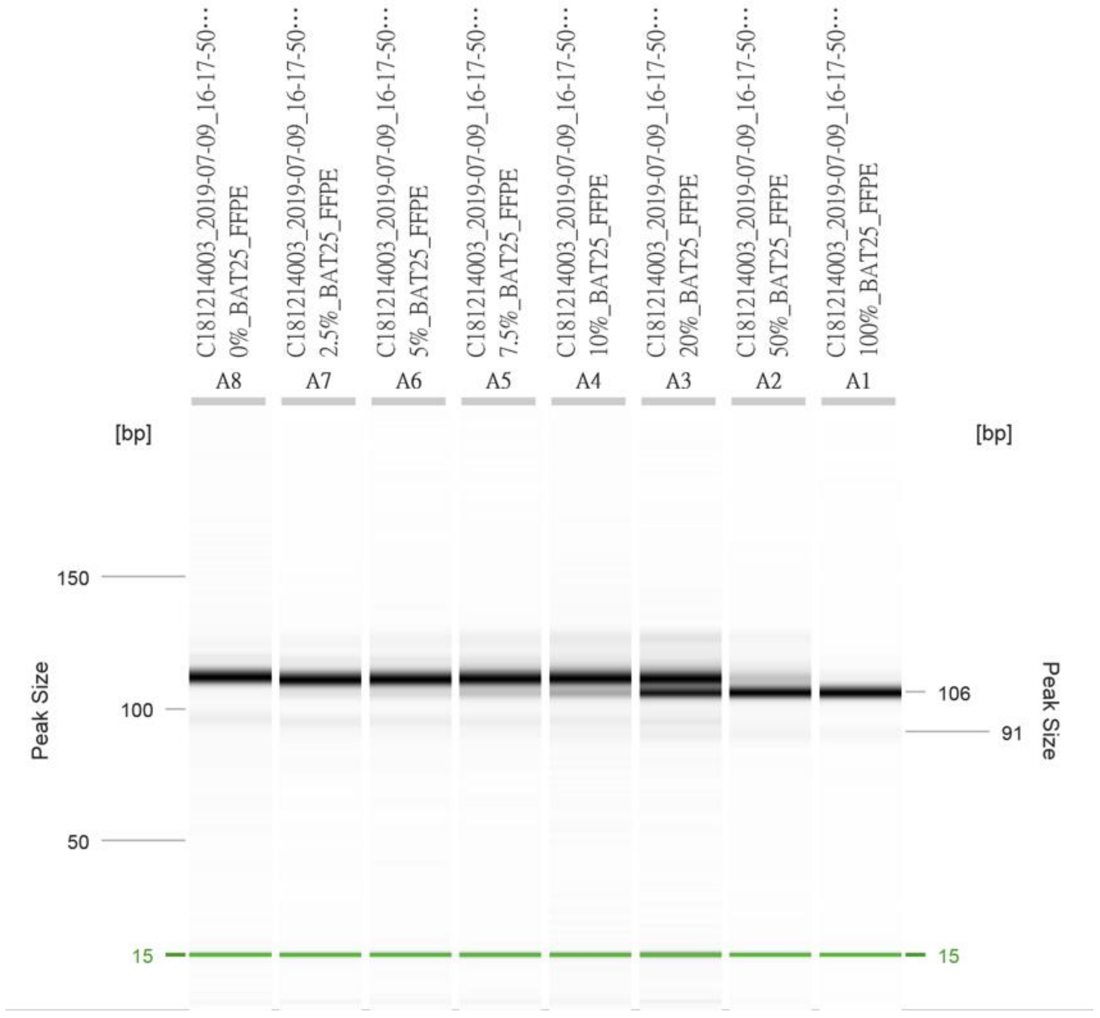

Figure 2 A FFPE BAT-25 high resolution gel original image (Right side)

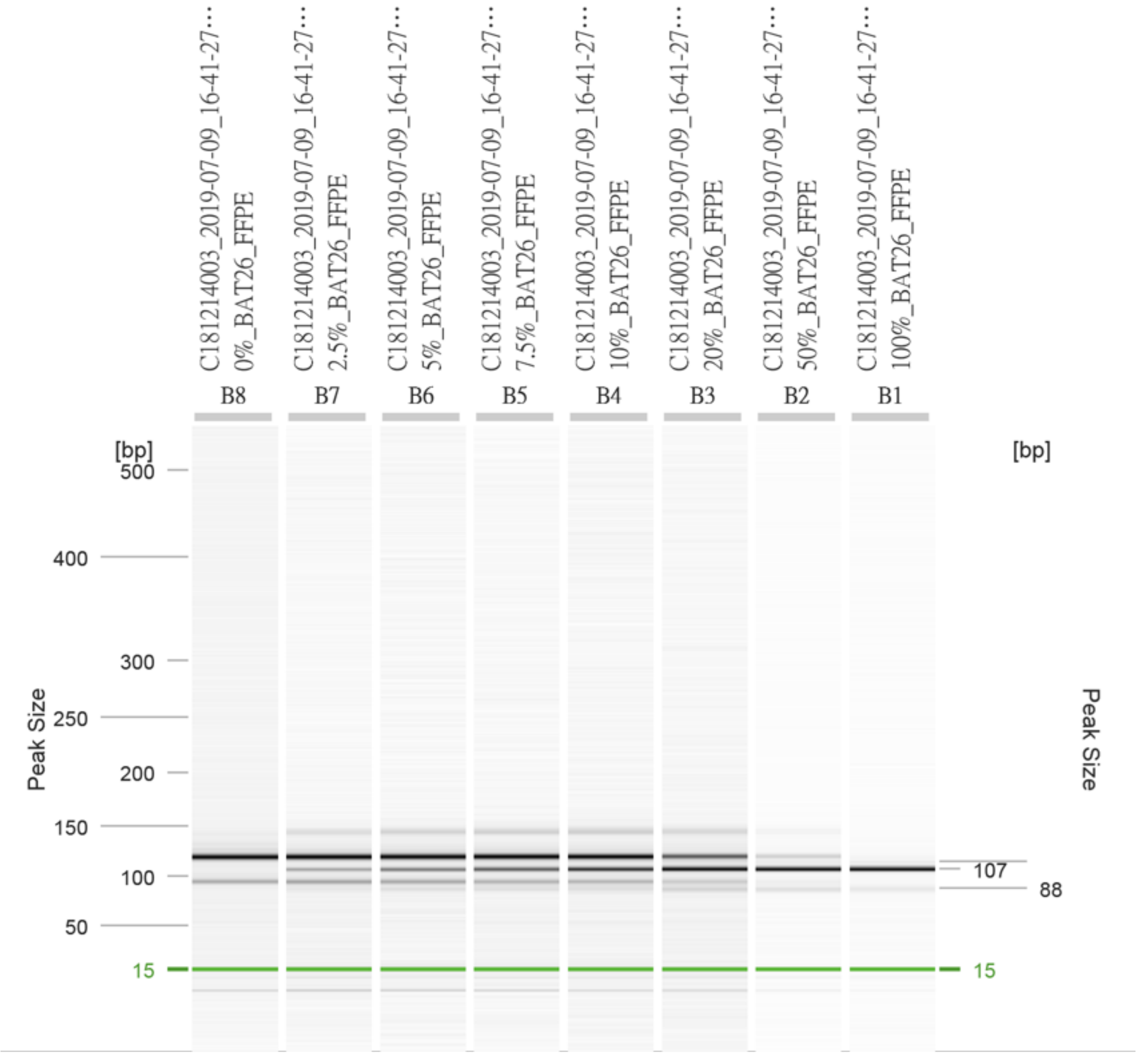

Figure 2 A FFPE BAT-26 high resolution gel original image (Right side)

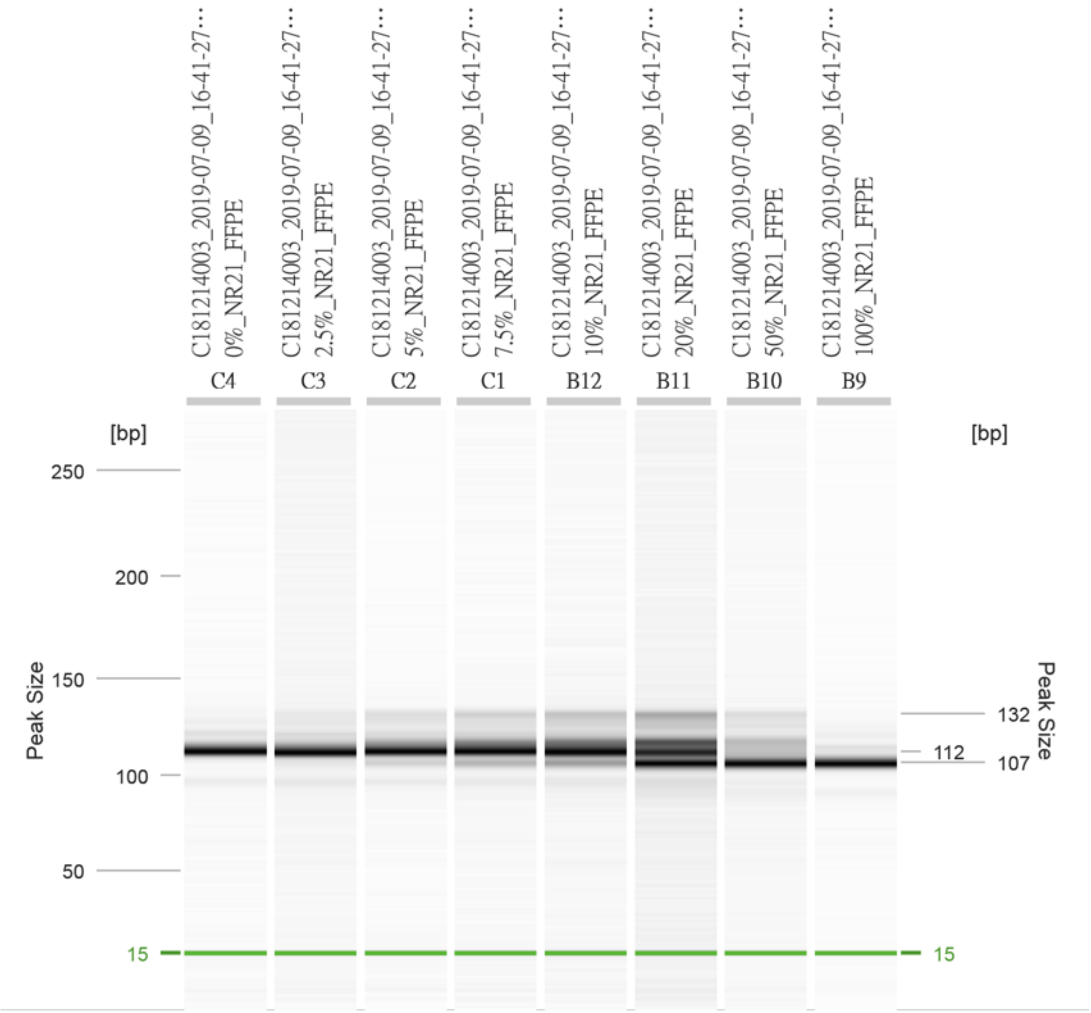

Figure 2 A FFPE NR-21 high resolution gel original image (Right side)

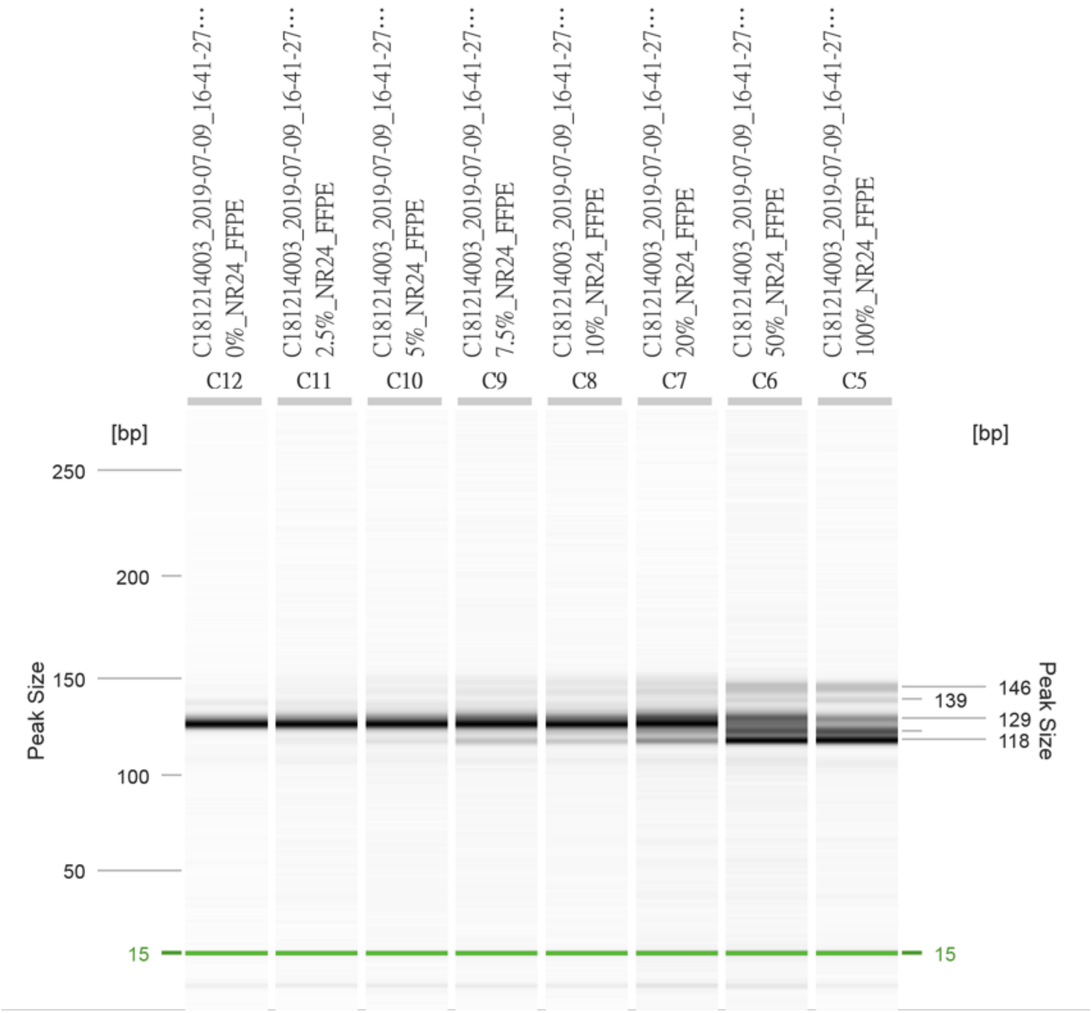

Figure 2 A FFPE NR-24 high resolution gel original image (Right side)

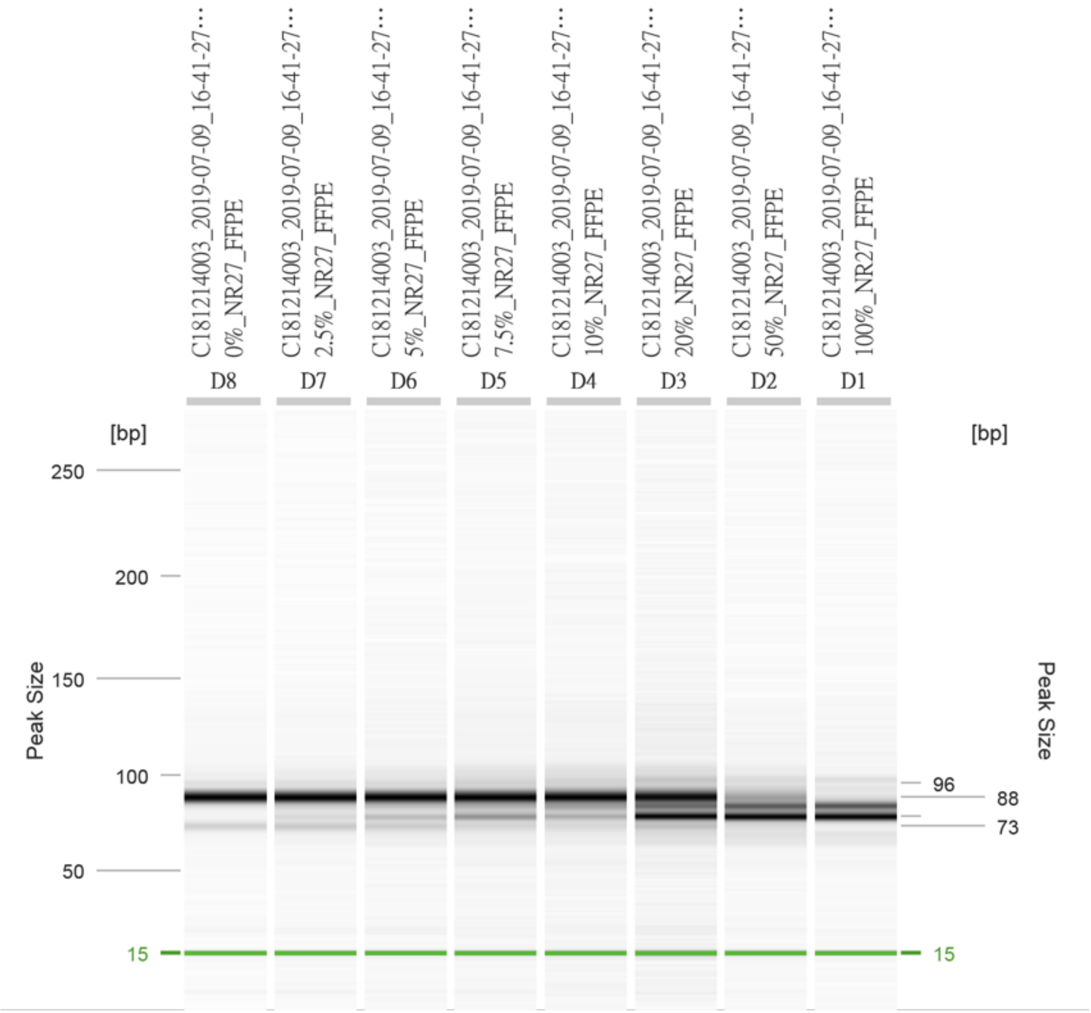

Figure 2 A FFPE NR-27 high resolution gel original image (Right side)

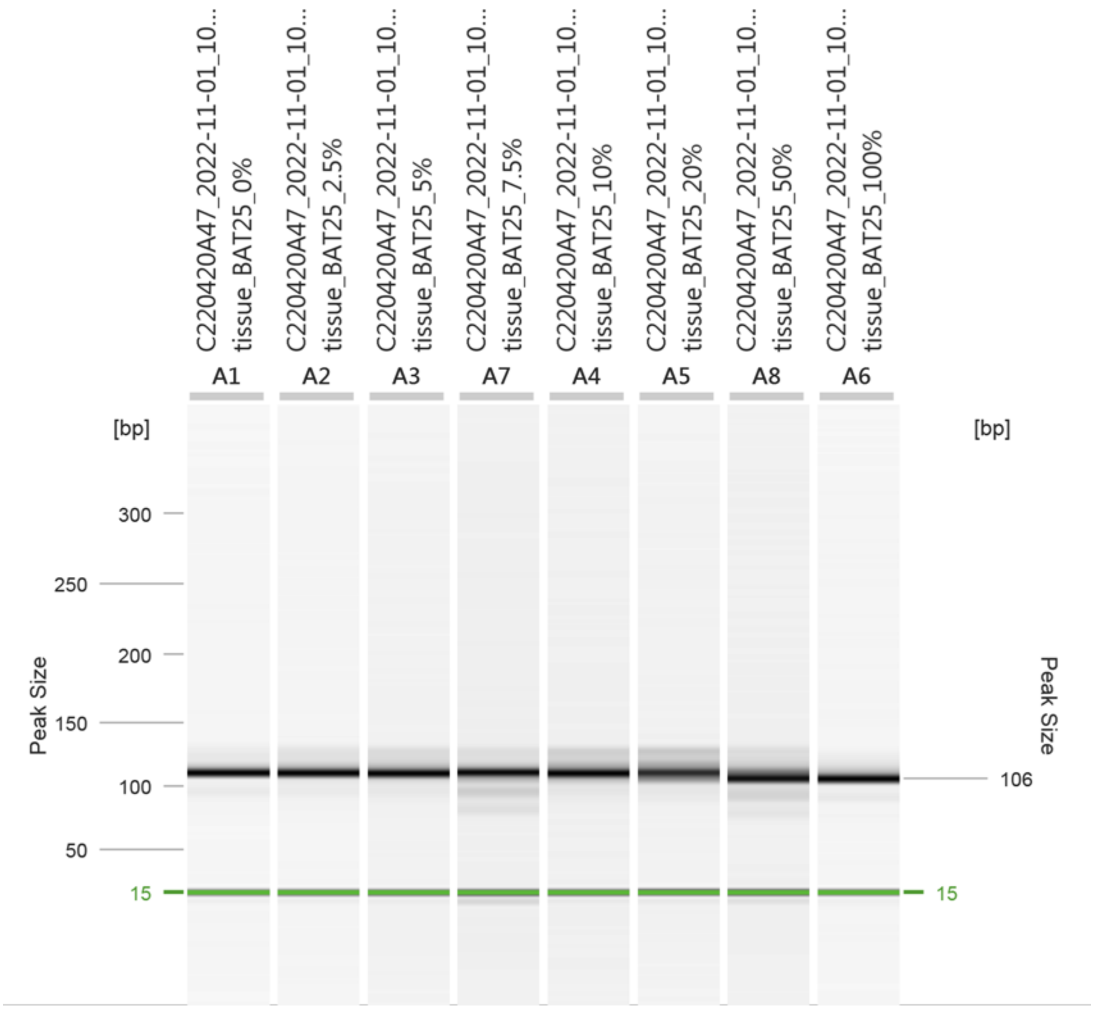

Figure 2 B Fresh frozen tissue BAT-25 Screen gel original image (Left side)

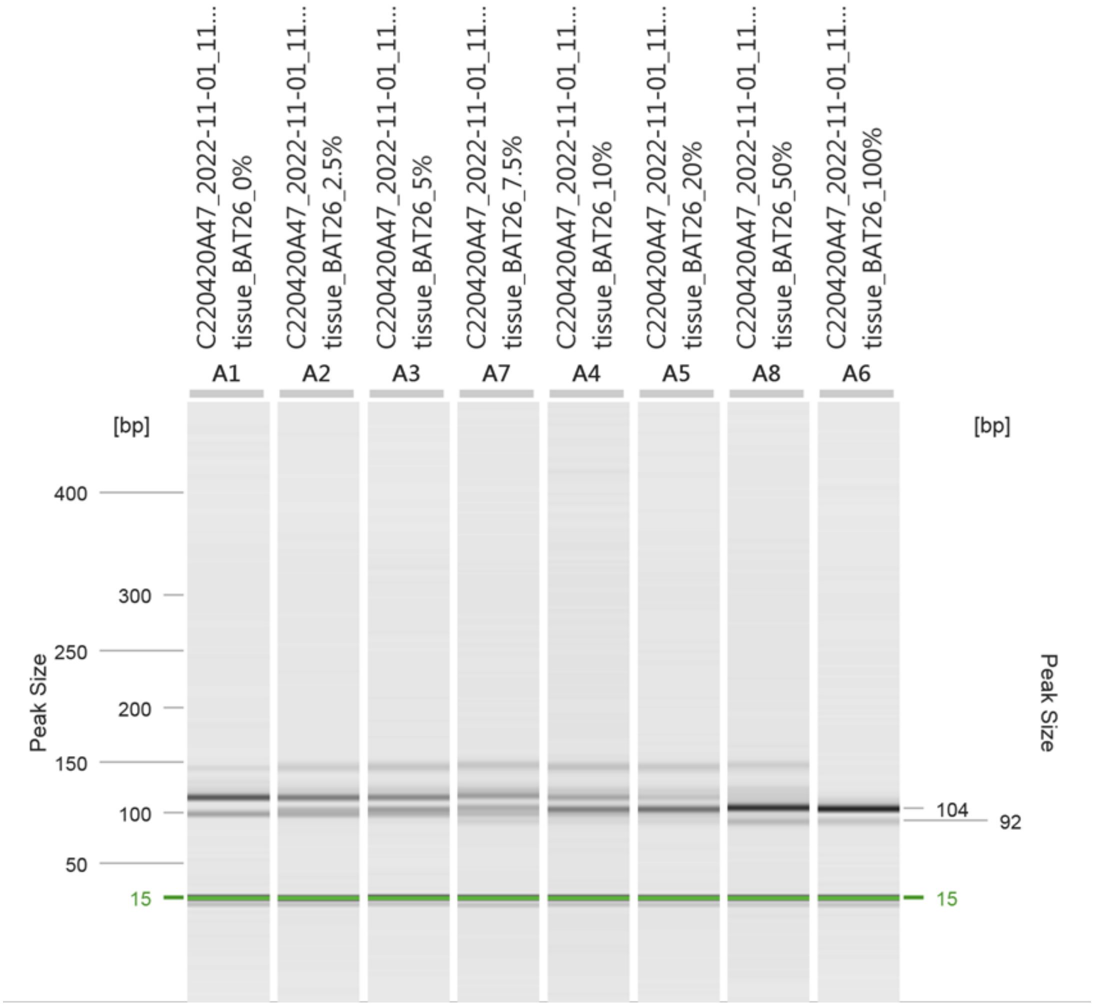

Figure 2 B Fresh frozen tissue BAT-26 Screen gel original image (Left side)

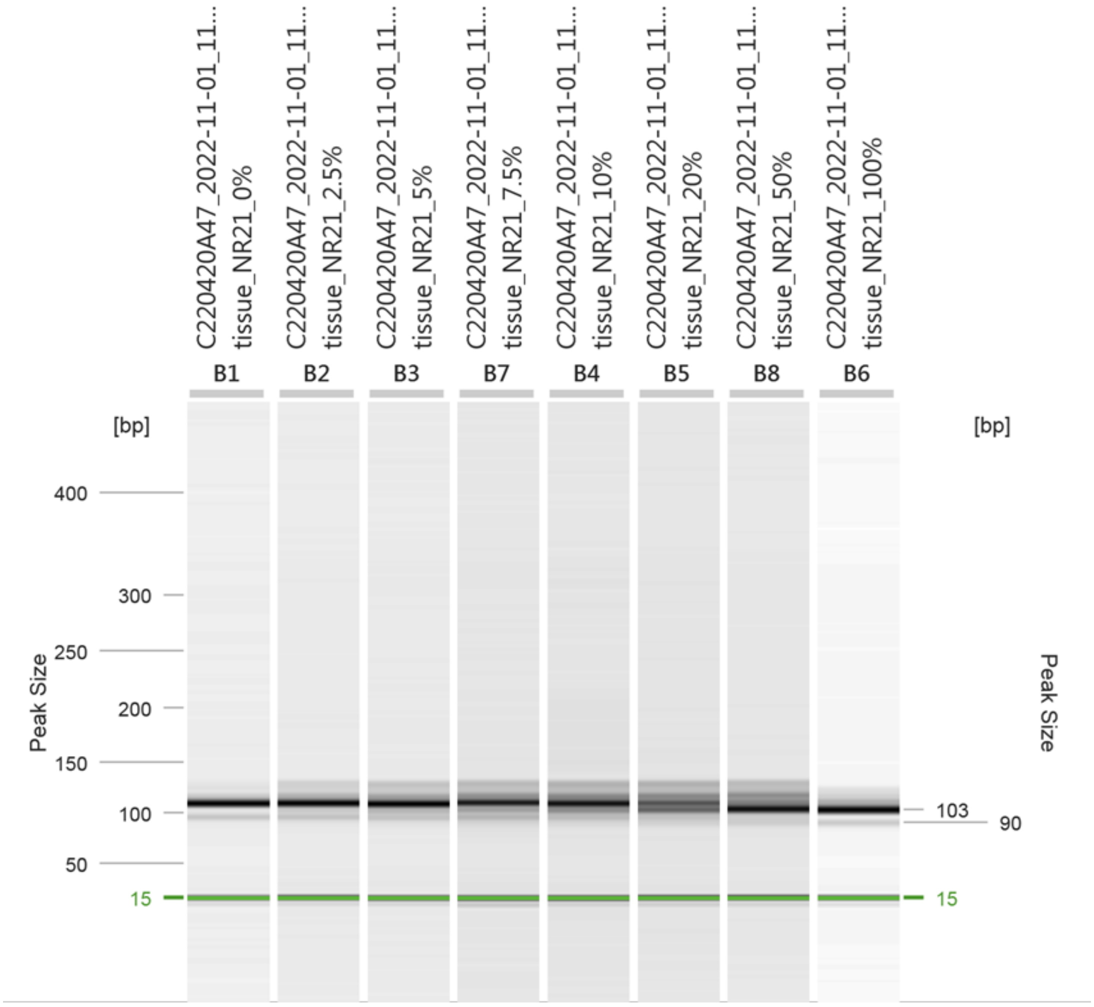

Figure 2 B Fresh frozen tissue NR-21 Screen gel original image (Left side)

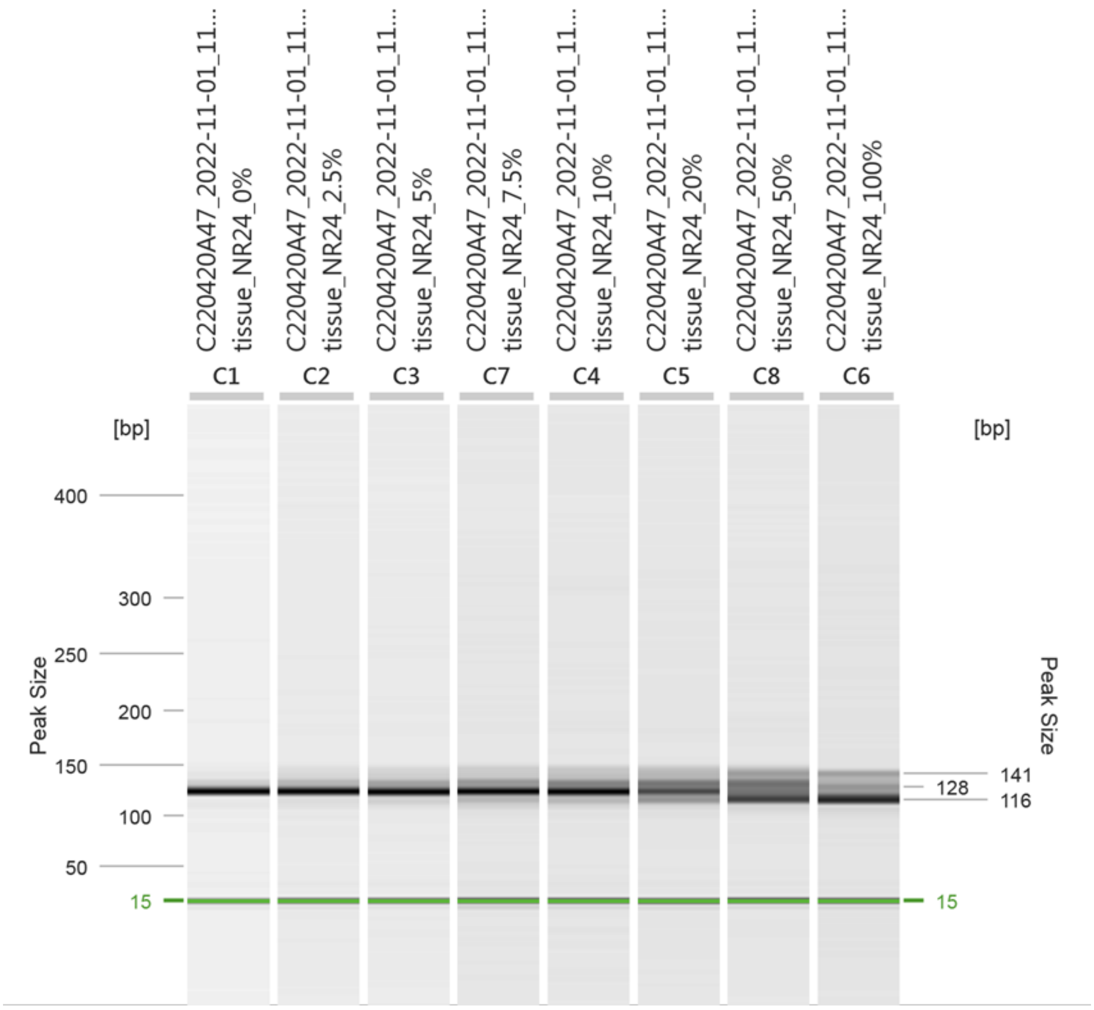

Figure 2 B Fresh frozen tissue NR-24 Screen gel original image (Left side)

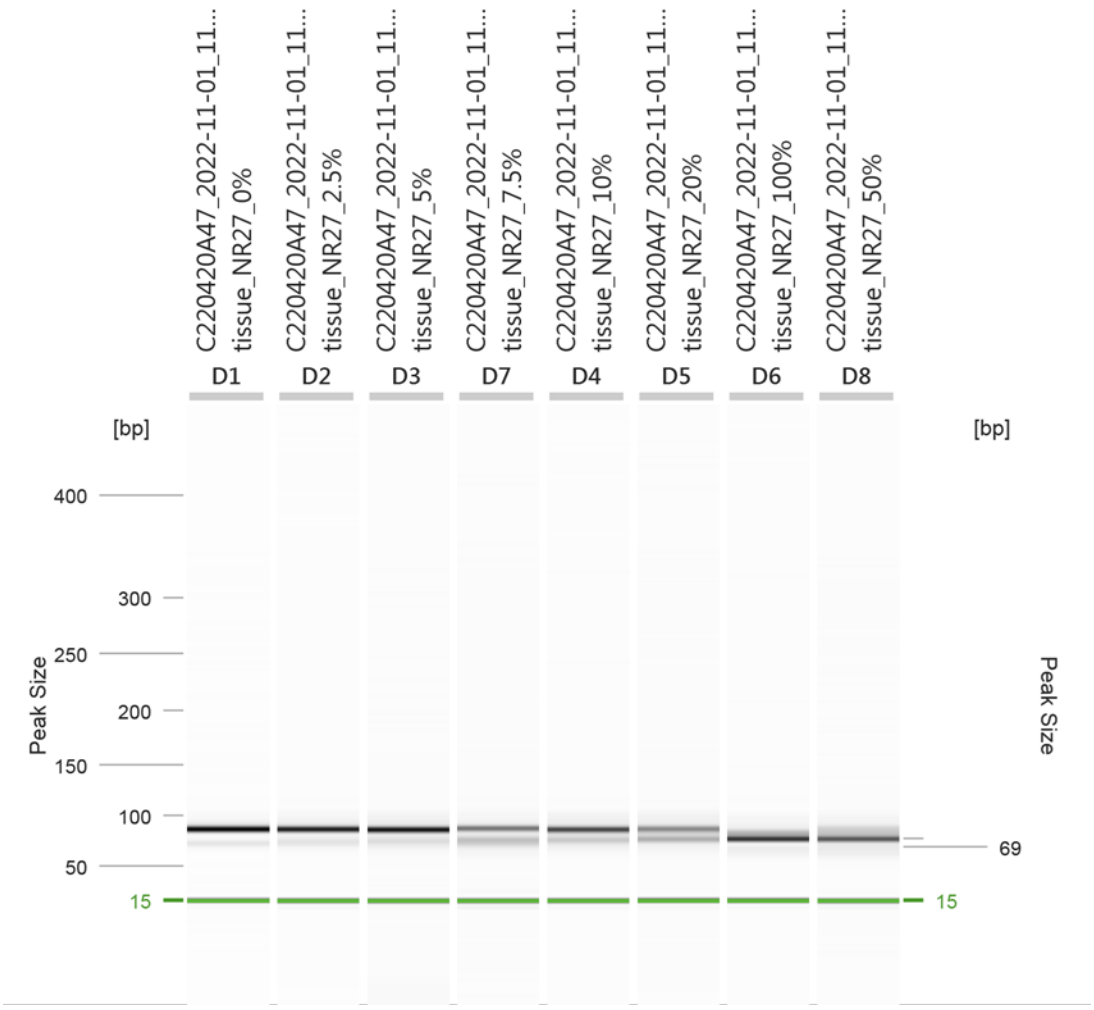

Figure 2 B Fresh frozen tissue NR-27 Screen gel original image (Left side)

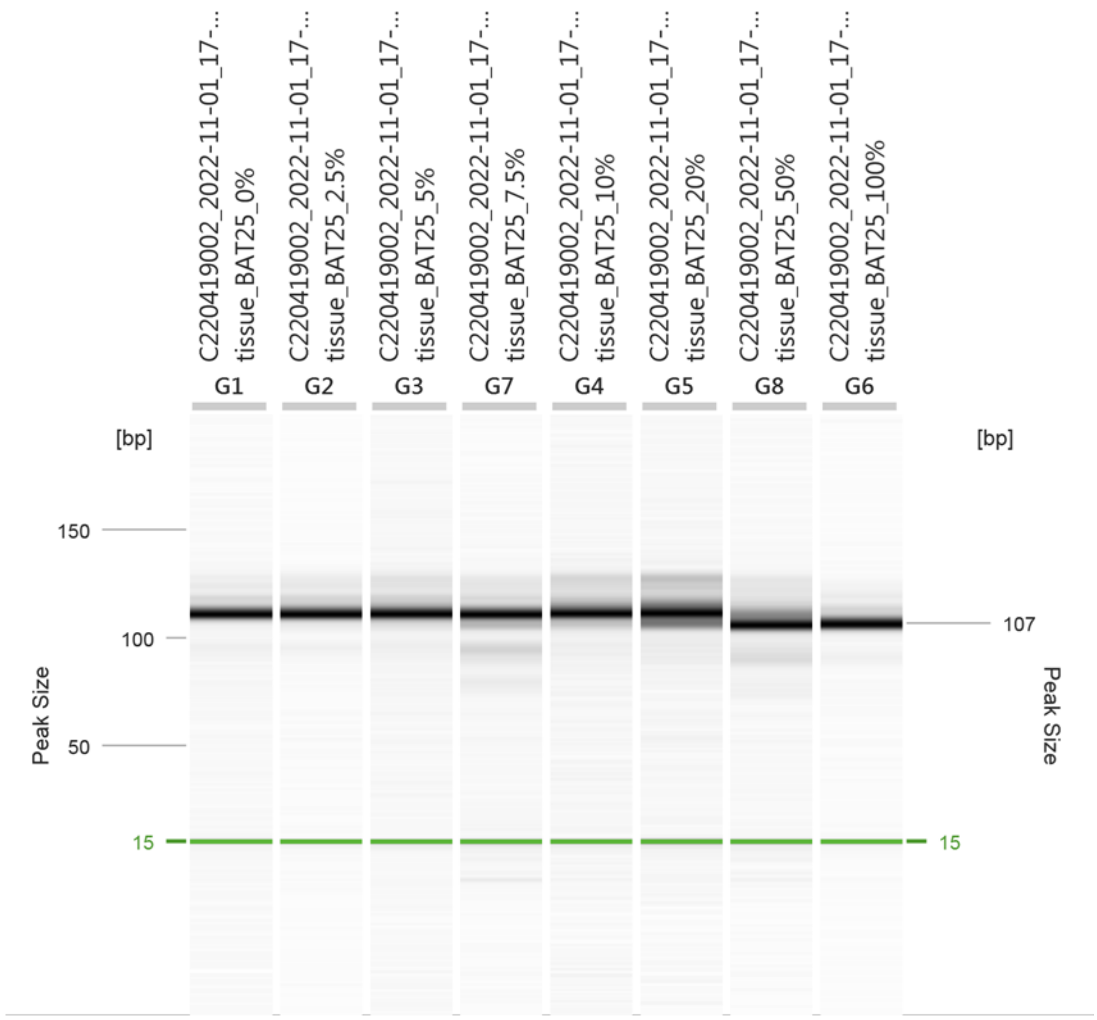

Figure 2 B Fresh frozen tissue BAT-25 high resolution gel original image (Right side)

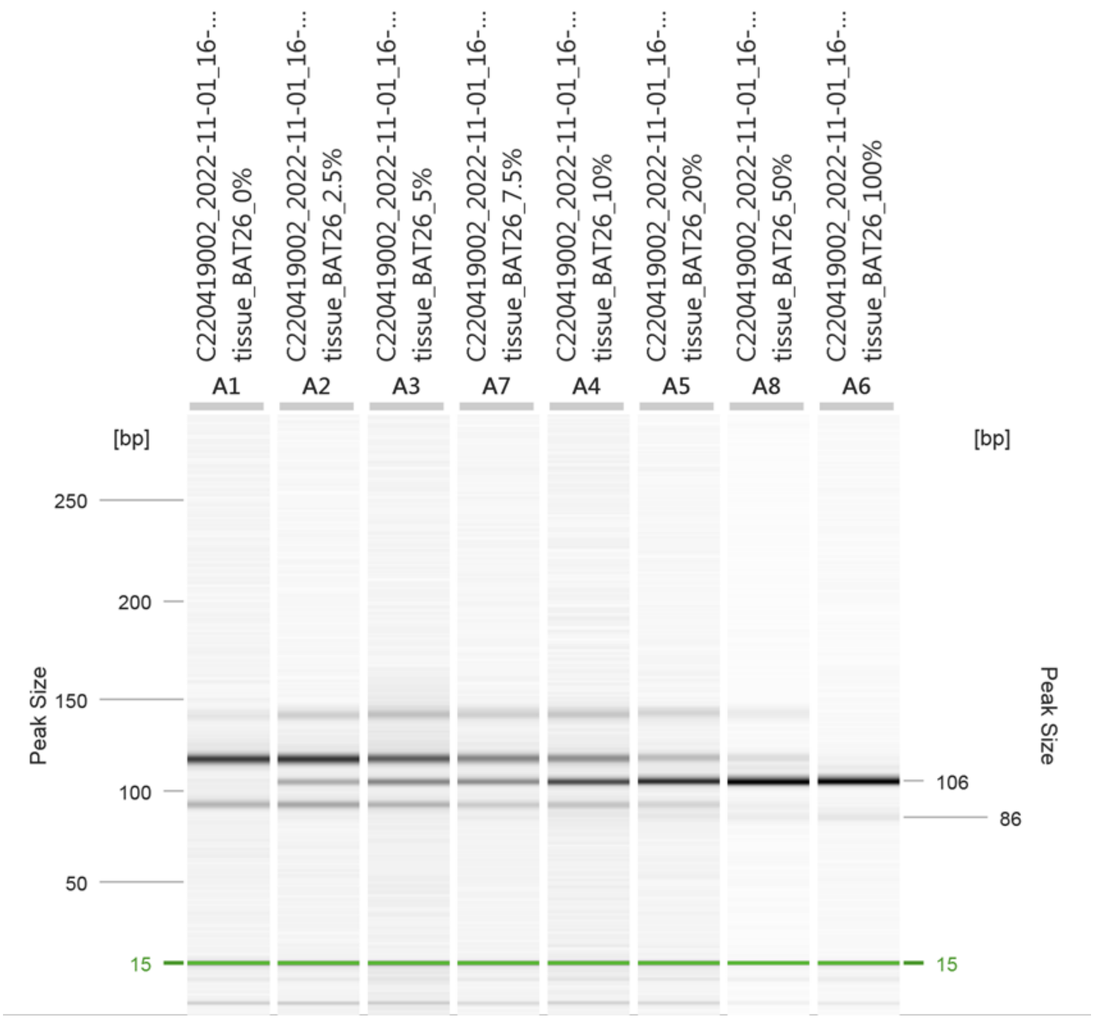

Figure 2 B Fresh frozen tissue BAT-26 high resolution gel original image (Right side)

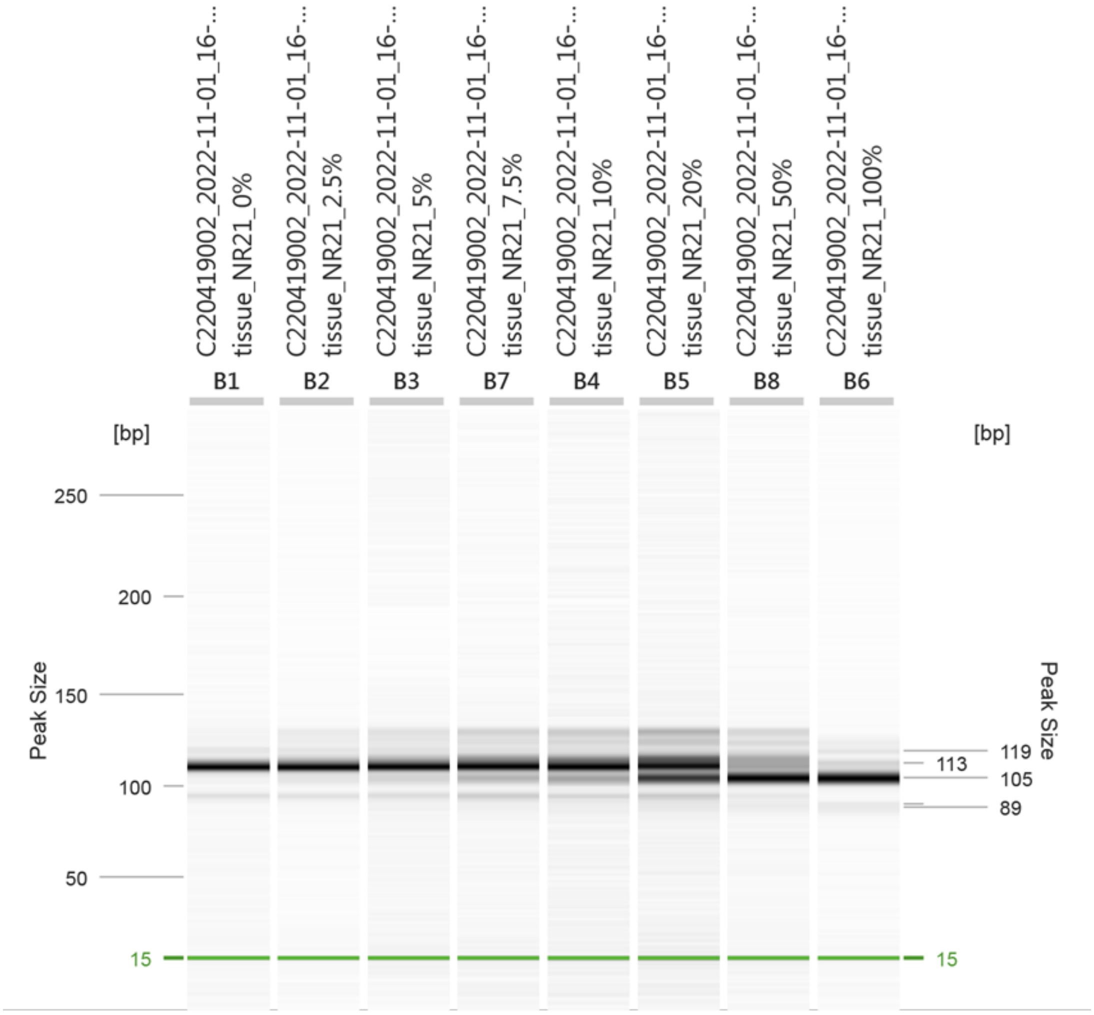

Figure 2 B Fresh frozen tissue NR-21 high resolution gel original image (Right side)

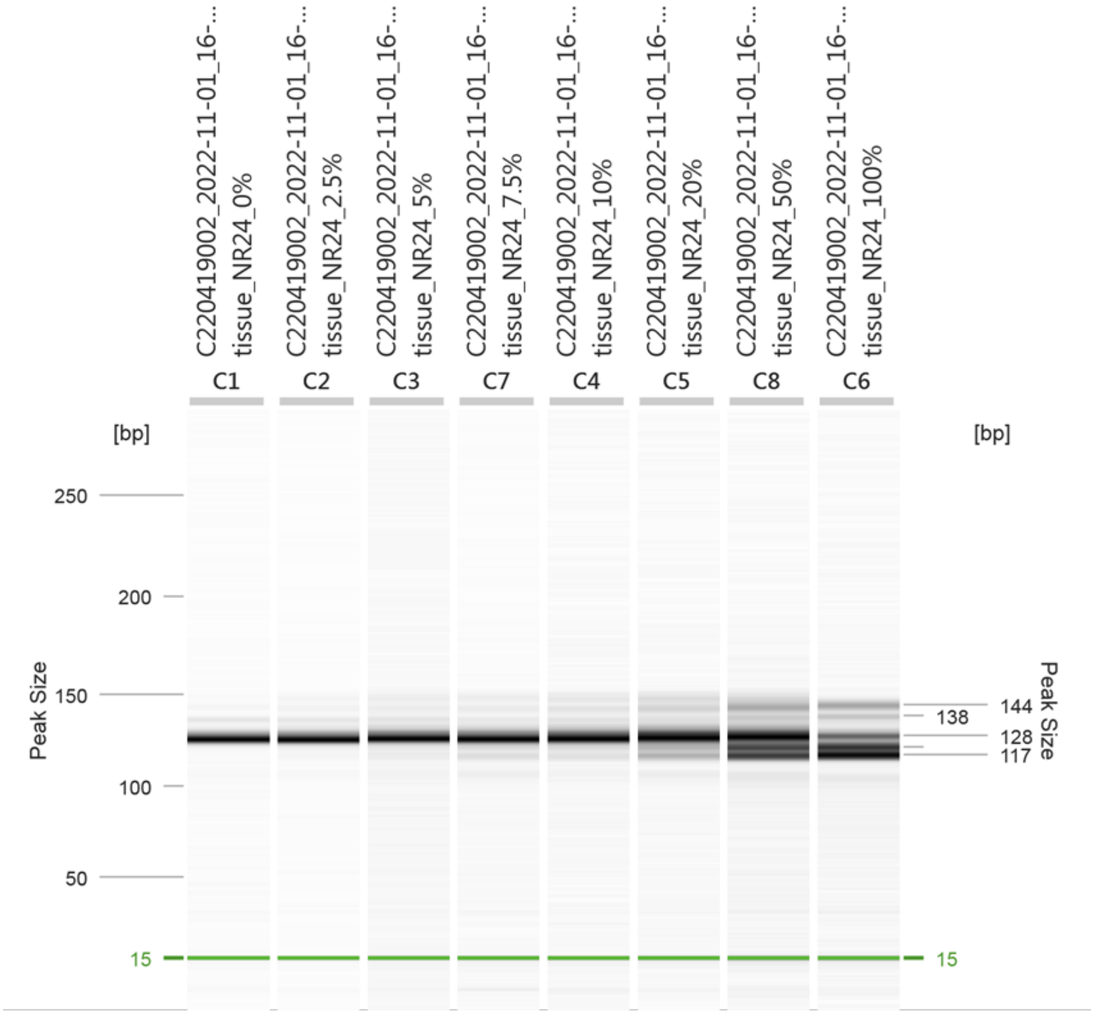

Figure 2 B Fresh frozen tissue NR-24 high resolution gel original image (Right side)

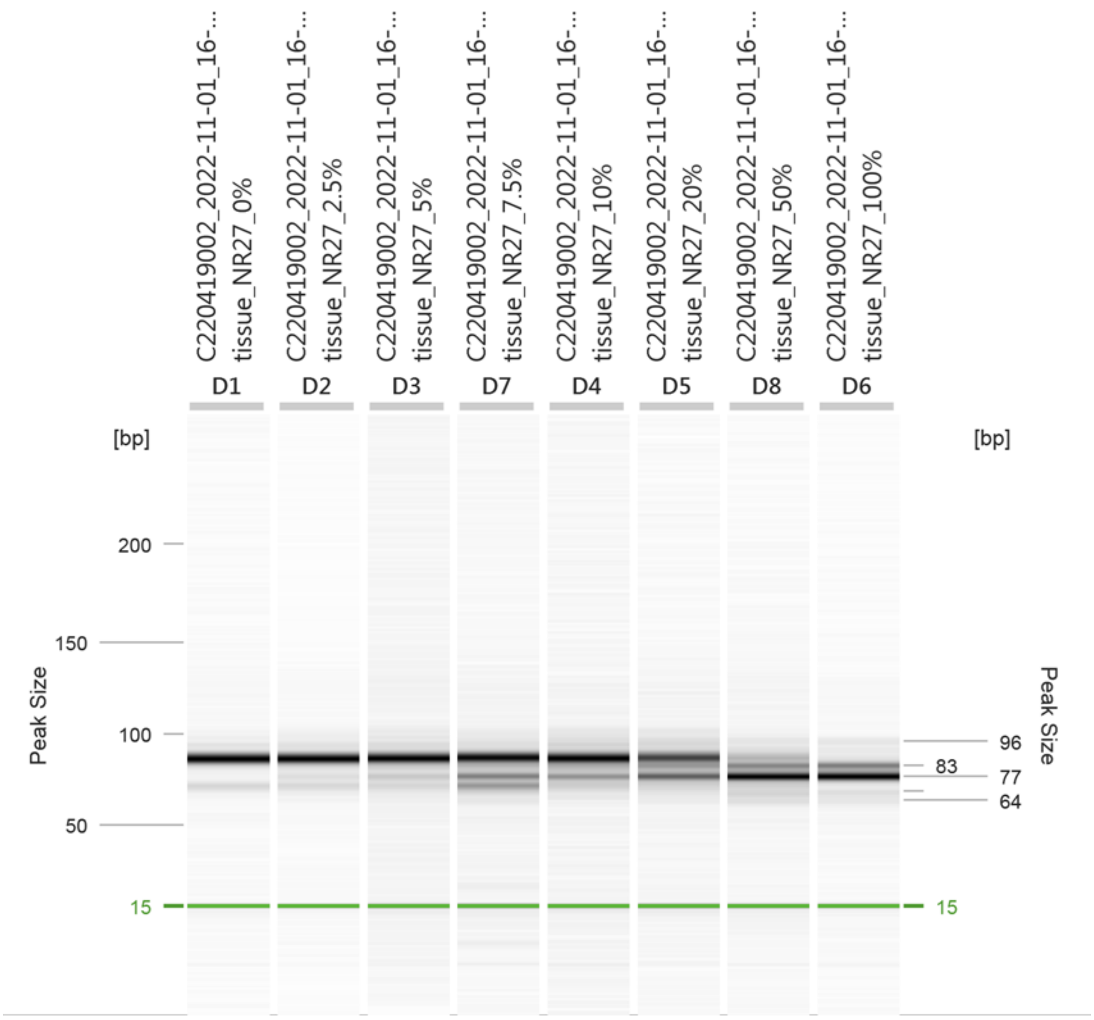

Figure 2 B Fresh frozen tissue NR-27 high resolution gel original image (Right side)

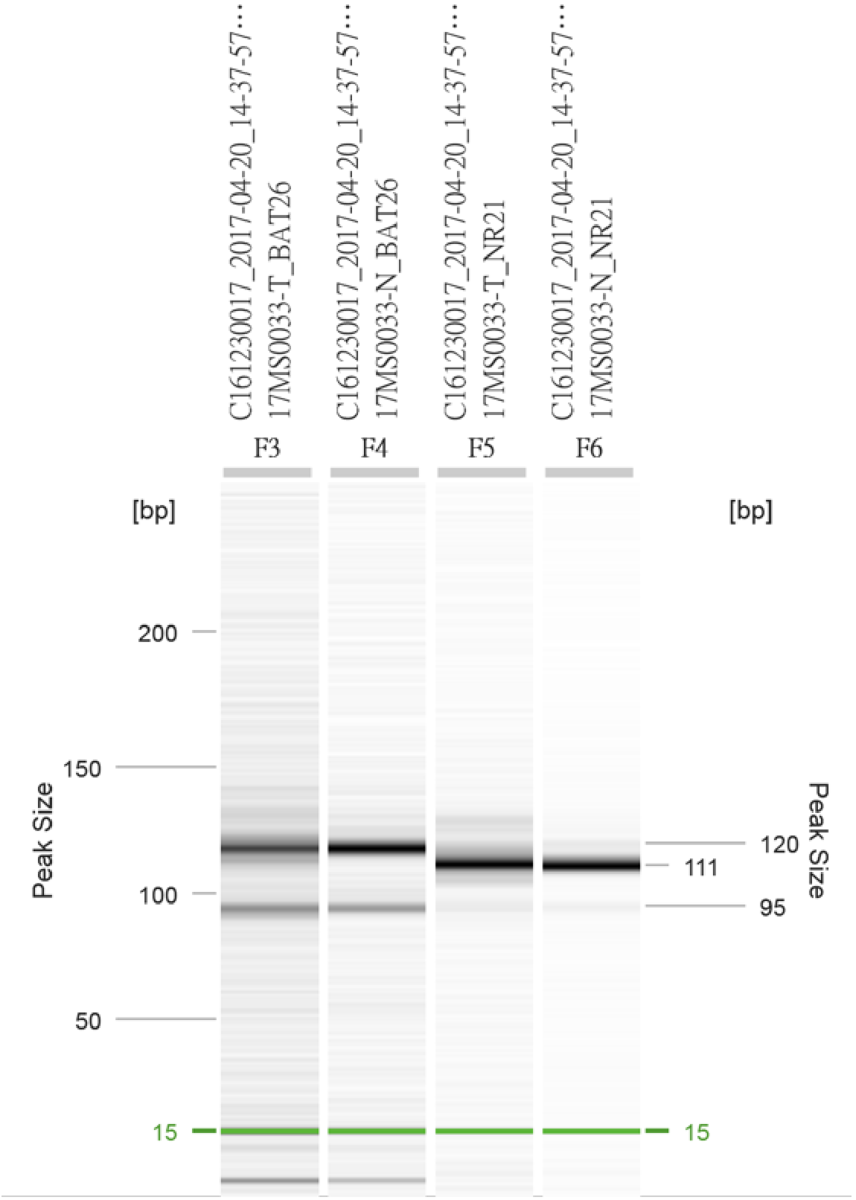

S2 Figure BAT-26 and NR-21 in screen gel original image

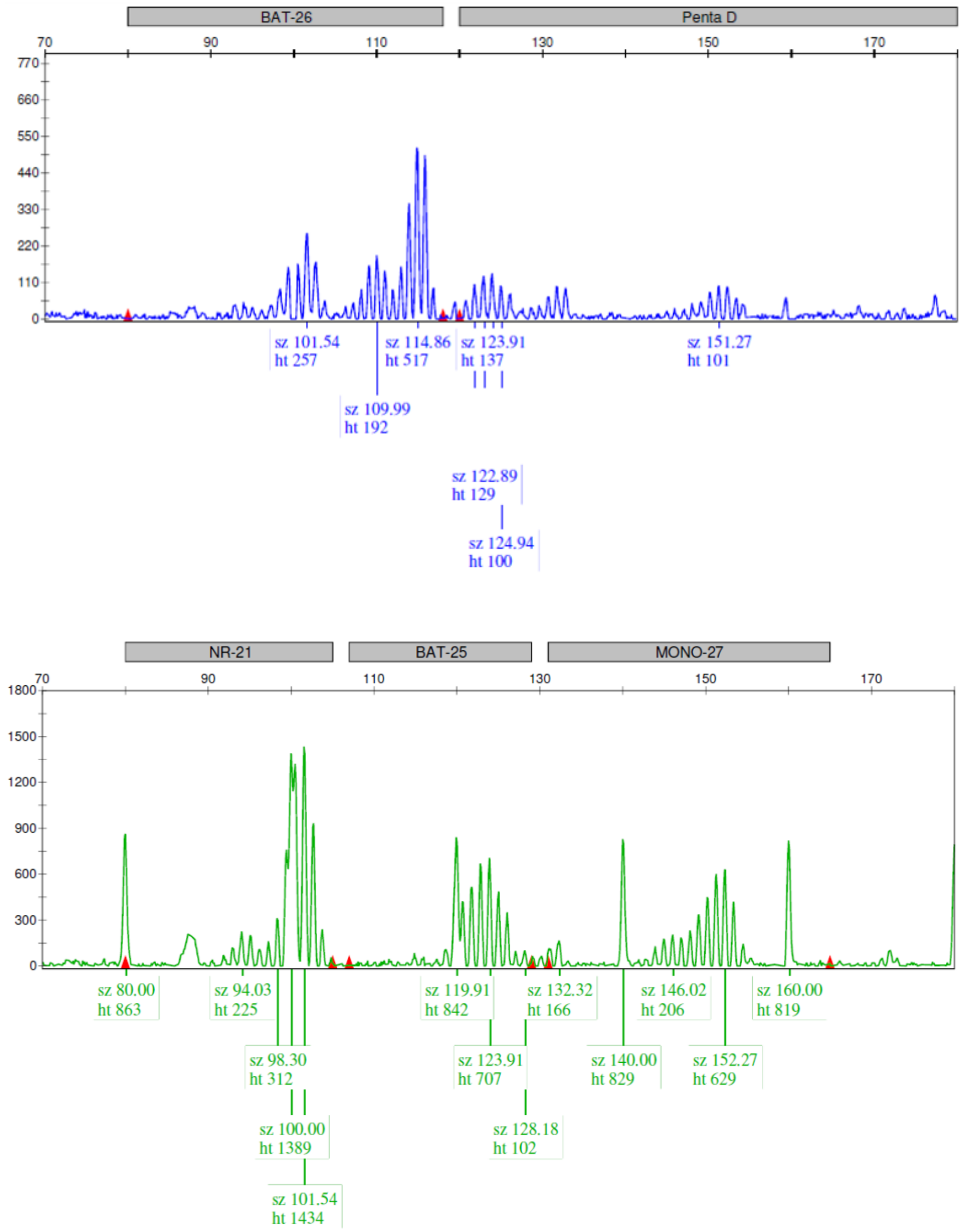

S2 Figure BAT-26 and NR-21 in GeneScan original image
